# Establishment and validation of a prognostic model based on vasculogenic mimicry-related gene clustering in ovarian cancer

**DOI:** 10.3389/fonc.2025.1575694

**Published:** 2025-09-25

**Authors:** Xueyuan Zhao, Yan Jia, Weijia Wen, Caixia Shao, Qiaojian Zou, Linna Chen, Hongye Jiang, Guofen Yang, Wei Wang, Chunyu Zhang, Shuzhong Yao

**Affiliations:** ^1^ Department of Obstetrics and Gynecology, the First Affiliated Hospital, Sun Yat-sen University, Guangzhou, Guangdong, China; ^2^ Guangdong Provincial Clinical Research Center for Obstetrical and Gynecological Diseases, Guangzhou, Guangdong, China

**Keywords:** ovarian cancer (OC), vasculogenic mimicry (VM), prognostic model, immune microenvironment, therapeutic response

## Abstract

**Background:**

As a critical prognostic factor in ovarian cancer which is the most lethal gynecologic malignancy, vasculogenic mimicry (VM) has not been systematically incorporated into prognostic evaluation frameworks in ovarian cancer (OC). This underscores the necessity to develop and validate a gene subtyping-based prognostic model through comprehensive analysis of VM-related biomarkers.

**Methods:**

This study integrated multi-omics data from TCGA, GEO and GTEx, forming a primary set and an external validation cohort. Through literature mining, 28 VM-related genes were identified. Univariate Cox and LASSO regression distilled 9 genes as vasculogenic mimicry-related prognostic index (VMRPI), establishing a risk model validated by ROC and constructing a nomogram with clinical prognostic factors. Consensus clustering stratified patients into VM-high/-low subgroups. Multi-angle assessments connected risk scores with tumor mutational burden, immune infiltration, and chemotherapy sensitivity. Clinical validation encompassed IHC-PAS detection of VM structures in 36 HGSOC paraffin specimens and qRT-PCR confirmation of gene expression in matched frozen tissues.

**Results:**

vasculogenic mimicry-related genes (VMGs) exhibited differential expressions in HGSOC versus normal tissues, with consensus clustering stratifying 474 patients into prognostically distinct VM-high/low subgroups. Prognosis-associated DEGs (n=758) enriched in ECM-receptor and PI3K-AKT pathways. A 9-gene prognostic model demonstrated robust predictive accuracy. Risk scores correlated with immune infiltration and drug sensitivity. Multivariate-validated nomogram integrating clinical factors and risk scores achieved precise survival prediction. IHC-PAS confirmed VM structures, with VM-positive cases showing upregulated VMGs and VMRPIs.

**Conclusions:**

VMG-based stratification revealed distinct prognostic ovarian cancer subgroups and a 9-VMRPI demonstrated robust prognostic power with validated immune-microenvironment, drug-response associations, IHC-PAS staining, and qRT-PCR confirmation.

## Introduction

Ovarian cancer (OC), ranking as the third most prevalent malignancy in the female reproductive system following cervical cancer and endometrial cancers, has emerged as the leading cause of mortality in gynecological oncology due to challenges in early diagnosis and late-stage chemoresistance recurrence, earning its designation as the “silent killer” of gynecologic malignancies (1, 2). According to the latest Global cancer statistics (GLOBOCAN) data, 2023 witnessed 314,000 new global OC cases and 207,000 OC-related deaths, accounting for 3.7% and 4.7% of total female cancer incidence and mortality respectively (3). Early-stage OC typically lacks pathognomonic clinical manifestations and is often incidentally detected via ultrasonography during routine examinations. Although early screening has not demonstrated prognostic improvement, interventions at this stage yield favorable outcomes with 5-year survival rates reaching 61-87% (4, 5). In contrast, advanced OC frequently presents non-specific symptoms including abdominal distension, palpable masses, ascites, and tumor burden or metastasis-related manifestations. Notably, 70% of patients are diagnosed at The International Federation of Gynecology and Obstetrics (FIGO) Stage III-IV due to its insidious onset and high metastatic propensity (1, 5). The characteristic tumor heterogeneity and reduced chemosensitivity in advanced cases contribute to a 5-year survival rate below 30% (5). Histologically, high-grade serous ovarian carcinoma (HGSOC) represents the most aggressive subtype, comprising 70-80% of OC cases as per World Health Organization (WHO) classification (6). HGSOC’s clinical aggressiveness and distinct biological features not only pose significant threats to women’s health but also establish it as a primary research focus in OC pathogenesis and therapeutics (6, 7). Current standard-of-care combining cytoreductive surgery with platinum-based multi-agent chemotherapy achieves short-term remission but faces limitations with 80% of patients developing platinum-resistant recurrence (7, 8). Emerging molecular strategies incorporating poly-ADP-ribose polymerase (PARP) inhibitors, anti-angiogenics and combination therapies are reshaping first-line maintenance approaches (9, 10). However, compensatory vascular endothelial growth factor (VEGF) pathway activation and tumor microenvironment remodeling underlie anti-angiogenic resistance, maintaining median survival at 12–18 months in platinum-resistant recurrent cases (8, 11, 12). The contemporary management of OC confronts dual challenges: persistent high recurrence rates with platinum resistance in advanced disease, and the urgent need for precision prognostic models to guide maintenance therapies in the molecular-targeted era (9, 12, 13). Traditional prognostic parameters prove inadequate for molecularly-driven precision medicine. Integrated prognostic models incorporating molecular markers with clinical variables could enable pretreatment outcome prediction and therapeutic decision-making, while multi-omics-based stratification may optimize maintenance therapy selection and duration (14, 15). Consequently, developing clinically translatable biomarker panels with prospective validation cohorts represents a critical frontier in advancing OC precision medicine paradigms.

Vasculogenic mimicry (VM) refers to a phenomenon wherein malignant solid tumor cells acquire endothelial-like phenotypes through phenotypic plasticity, autonomously forming functional three-dimensional tubular networks without endothelial cell participation. These VM channels substantially enhance nutrient and oxygen supply to tumor cells located either within the tumor mass or distal to capillary beds (16, 17). First documented by Maniotis et al. in 1999 in aggressive uveal melanoma, this discovery fundamentally challenged conventional tumor angiogenesis paradigms, offering novel insights into the “tumor cell-driven” vascularization mechanism underlying malignant blood supply patterns (17). Subsequent studies have identified VM structures in 24 distinct malignancies including breast cancer, non-small cell lung carcinoma, and glioblastoma, with their presence correlating with enhanced nutrient acquisition and metastatic potential (16, 18–21). Current mechanistic studies generally propose that specific cell subpopulations with differentiation potential within tumors, under conditions of oxygen and nutrient deprivation, induce and activate multiple pathways to transdifferentiate into tumor cells with endothelial-like phenotypes. These cells then form vascular-like channels through intercellular connections, with subsequent blood perfusion completing VM structure formation (22–25). The molecular mechanisms underlying this structure involve multi-dimensional regulatory networks: (1) In microenvironmental stress: Hypoxia-induced HIF-1α or HIF-2α drives tumor cell metabolic reprogramming through activation of the PI3K/AKT/mTOR signaling axis; (2) In cellular plasticity: The epithelial-mesenchymal transition (EMT) process facilitates the acquisition of cancer stem cell characteristics; (3) In structural remodeling: The VE-cadherin/EphA2/MMPs pathway promotes lumen formation by regulating intercellular junction complex assembly and basement membrane metabolism and remodeling (26–30). Notably, beyond these three pathways, other genes including ALDH1A1, BCAR3, CGB5, MIR27B, MIR765, PLAU, SEMA4D, XAF1, and TCF4 also play significant roles in VM formation (22, 31–34).

Research on VM structures in ovarian cancer has also been documented. The work of Jing Du et al. demonstrated that hypoxic microenvironment could promote VM formation by inducing EMT in ovarian cancer cells, elucidating the pathway mechanisms underlying VM existence in OC (25). Furthermore, substantial studies have confirmed that VM-positive ovarian cancer patients exhibit significantly shorter overall survival and higher metastatic incidence compared to VM-negative counterparts (35). Although significant progress has been made in understanding VM molecular mechanisms in recent years, most studies remain fragmented at the molecular level, and its potential as a prognostic factor remains under-explored in survival assessments (35–37). Current multi-omics prognostic models for ovarian cancer predominantly focus on endothelial-dependent angiogenesis in blood supply mechanisms, while neglecting VM - an autonomous tumor cell-driven vascular system. This oversight has led to the long-term exclusion of this critical biological feature from prognostic frameworks. This study aims to distinguish subgroups with significant prognostic differences based on vasculogenic mimicry-related genes (VMGs), subsequently revealing inter-subgroup heterogeneity in gene expression through screening of differentially expressed genes (DEGs) combined with differential expression analysis and functional enrichment studies. Prognostically significant DEGs between subgroups will then be selected to construct a vasculogenic mimicry-related prognostic index (VMRPI) as a prognostic model, which will be rigorously validated. The model is designed not only to effectively predict survival outcomes in ovarian cancer patients, but also to characterize immune microenvironment features and chemotherapy drug sensitivity across different risk subtypes. Finally, experimental validation will be conducted to identify VM structures in our institutional patient cohort and verify the expression differences of VMRPI components.

## Materials and methods

### Data collection and processing

This study collected RNA sequencing data and clinical information of 429 HGSOC cases from The Cancer Genome Atlas (TCGA, https://portal.gdc.cancer.gov) database, and identified all available HGSOC datasets from the Gene Expression Omnibus (GEO, https://www.ncbi.nlm.nih.gov) database. The screening criteria focused on whether datasets contained VMGs and whether their sequencing results were presented in transcripts per million (TPM) format to ensure optimal compatibility with TCGA data. Ultimately, 98 HGSOC samples with clinical information from dataset GSE51088 were selected. These two datasets were merged using bioinformatics methods to form the training set and internal validation set, collectively defined as the primary dataset. Additionally, RNA sequencing data and corresponding clinical information from 110 HGSOC patients in GSE17260 of GEO were obtained to construct the external validation dataset. Considering current omics analyses and substantial evidence indicating that HGSOC likely originates from fallopian tube epithelium, normal control samples utilized RNA sequencing data from 180 normal fallopian tube epithelial tissues obtained through the Genotype-Tissue Expression (GTEx, https://www.gtexportal.org) portal. All data from TCGA, GEO, and GTEx databases were publicly accessible and strictly complied with data acquisition and usage policies during their application.

After excluding data that did not meet the experimental criteria, performing pathological type screening, and processing the matched sample survival information of the afore-mentioned samples, 474 samples in the primary dataset retained clinical prognostic information and were utilized, while the external validation set contained 110 samples with corresponding prognostic information for external model validation. The clinical characteristics and data sources of patients in the study cohort are presented in **Supplementary Table S1**. The preprocessing of the data included data transformation and merging. First, for datasets requiring integration—specifically TCGA with GEO, and TCGA with GTEx—the “Combat” function from the “sva” package in R was employed to remove batch effects arising from different sequencing platforms. To analyze and validate whether the merged data from TCGA and GEO exhibited differences in distribution or batch effects, Principal Component Analysis (PCA) was performed using the “prcomp” function from the “stats” package in R, followed by visualization.

### Construction of protein-protein interaction network

The protein-protein interactions (PPIs) network was constructed using version 12.0 of the Search Tool for the Retrieval of Interacting Genes/Proteins (STRING, https://cn.string-db.org) database, a comprehensive repository of experimentally confirmed and computationally predicted protein interactions. This database is openly accessible and freely available for research purposes.

### Identification of prognostic VM-related genes

VM-related genes for ovarian cancer were identified through a comprehensive search of the literature published on PubMed over the past 20 years, including both primary research articles and review articles (22, 28, 31–33). This gene set is available in **Supplementary Table S2**. The univariate Cox analysis was utilized to identify VMGs, including SNAI1, MMP2, MMP14, SNAI2, ZEB1, and TWIST1, as the core genetic determinants using false discovery rate (FDR) <0.05 as the significance threshold. The R package “survival” was used for survival analysis. Kaplan-Meier estimation with optimal cutoff determination was performed for univariate assessment, and univariable Cox proportional hazards regression models were subsequently fitted to quantify the association between individual variables and survival outcomes. The “ggsurvplot” function generated survival curves visualizing associations between gene expression and clinical outcomes.

### Consensus clustering

To identify VM subtypes, a rigorous unsupervised classification algorithm, named consensus clustering analysis in R package “ConsensuClusterPlus”, was performed using the expression of the determinant core genes and Euclidean distance was set as 1000 times repetition (38). In the experiment, we aim to determine two values: the optimum cluster number (k) and the degree of consensus stability. Finding the output from the cumulative distribution function (CDF) plots and determining whether the CDF curve is flat is necessary for this. Computational optimization identified k=2 as the optimal partitioning threshold, resulting in division of the primary dataset into two subgroups: VM-high and VM-low. Prognostic disparities between subgroups were subsequently confirmed through statistical testing.

### Identification and analysis of differentially expressed genes

In the primary dataset, DEGs were identified and filtered from contrasting the risk-high and risk-low subgroups using the “limma” R package, with key parameters set as |fold change| >1.5 and a significance level of FDR <0.05. Volcano plots of DEGs were generated via the “ggplot2” package. DEGs were investigated to assess both Gene Ontology (GO), including molecular function (MF), biological process (BP) and cellular component (CC), and intracellular metabolic pathways from and gene functions Genes and Genomes (KEGG) enrichment analysis (39, 40).

### Construction and validation of the risk score prognostic model

There were 260 DEGs showed significant prognostic association with their expression from DEGs (n=758) identified between the clusters using the univariate Cox regression analysis when the level was set at p<0.05. In the primary set, 4/5 of the patients were divided into the training set while 1/5 samples were divided into the internal validation by the “createDataPartition” function in the “caret” R package. The least absolute shrinkage and selection operator (LASSO) penalized Cox regression analysis was employed to reduce the candidate gene pool for developing the prognostic model by using the R package “glmnet”. The penalty parameter (λ) was determined using a ten-fold cross-validation approach, selecting the value that met the minimum criteria. After calculations, 9 genes were identified to construct the prognostic signature. The risk score model, named Risk Score, was established using β (coefficient) value multiplied by the expression of risk genes. The risk score formula was as follows: Risk Score = (β1*Exp1+β2*Exp2+ … +βn*Expn). The effectiveness of the model was evaluated through the R packages “survival”, “survminer”, and “timeROC” by performing receiver operating characteristic (ROC) curves and calculating the Area Under the Curve (AUC) values. Additionally, the ROC-based AUC evaluation method was similarly performed in the external validation set.

### Mutation profile analysis

Genomic somatic mutation profiles of the cohort were retrieved from the TCGA portal. Risk stratification was performed by dichotomizing patients at the median risk score into distinct high- and low-risk subgroups. Comparative analysis of mutation patterns across subgroups was visualized through waterfall plots constructed using the “maftools” package in R, as detailed in reference (41).

### Independent prognostic analysis and nomogram construction

In the primary dataset, an independent prognostic examination of the samples’ clinical features (e.g., age, tumor grade, and tumor stage) as well as risk scores was conducted via univariate Cox regression analysis to construct a nomogram model. The coefficients within the nomogram were derived using multivariate Cox regression analyses. The construction of the nomogram was performed using the “coxph” function from R packages, and a visual plot was generated via the “regplot” function.

Utilizing the “rms” and “survival” R packages, a nomogram model based on a multivariate Cox proportional hazards regression was established, incorporating age, tumor grade, tumor stage, and the Risk Score index. This model aims to quantitatively predict patients’ overall survival probabilities at 1-, 3-, and 5-year intervals. Calibration curves were also generated to visually evaluate the predictive accuracy of prognosis outcomes during the nomogram development process.

### Evaluation of microenvironment and cell infiltration

To reveal the relationship between the immune microenvironment, immune cell infiltration with risk score stratification in HGSOC patient samples, the R package “estimate” was used to calculate stromal, immune, and ESTIMATE scores. The CIBERSORT algorithm was utilized to quantitatively analyze the relative abundance of 20 immune cell types derived from the TCGA dataset, aiming to demonstrate the association between different immune cell subtypes and VMRPI. The principle of CIBERSORT lies in its use of RNA sequencing data to evaluate the enrichment level of specific cell types in mixed tissue samples, which is frequently applied to calculate immune cell infiltration within the tumor microenvironment (42). To investigate the association between immune checkpoints and risk scores, we employed a curated set of 79 immune checkpoint genes (ICGs) as our analytical framework (**Supplementary Table S3**) (43). These ICGs, systematically compiled from published literature, were analyzed to examine their differential expression patterns between high- and low-risk subgroups. Statistical values were obtained through Spearman correlation analysis, and heatmaps were generated using the “limma” and “ggplot2” packages.

### Drug sensitivity

Drug sensitivity prediction employed the algorithm framework of the R package “oncoPredict” which utilizes data from the Cancer Therapeutics Response Portal (CTRP, https://portals.broadinstitute.org/ctrp) and Genomics of Drug Sensitivity in Cancer (GDSC, https://www.cancerrxgene.org) databases, including TPM gene expression data from 28 HGSOC -related cell lines and IC_50_ values of 497 drugs, to construct a drug sensitivity prediction model based on ridge regression and provide predictive outcomes (44). The modeling process incorporated key preprocessing steps including log_2_ transformation, Empirical Bayes (ComBat) batch effect correction, and low-variance gene filtering. Using this algorithm, the therapeutic efficacy of commonly used chemotherapy drugs and gene-targeted therapies was predicted, and their IC_50_ values were calculated. These values were compared between high- and low-risk score groups, with box plots generated for visualization.

### Clinical specimens

Clinical samples were collected from patients who underwent primary treatment in the Department of Gynecology at The First Affiliated Hospital of Sun Yat-sen University between June 2023 and December 2023. A total of 36 patients who met the inclusion criteria, did not meet the exclusion criteria, and had matched paraffin-embedded sections and frozen tissues were included.

Inclusion criteria: (1) Patients who was diagnosed ovarian cancer according to the 2022 edition of the Chinese Guidelines for the Diagnosis and Treatment of Ovarian Cancer; (2) Aged between 18 and 75 years old; (3) Enrolled cases must present with a histologically confirmed primary tumor that fulfills the specified criteria; (4) Adequate other organ and bone marrow function; (5) No history of other malignant tumors. Exclusion criteria: (1) Individuals with severe underlying diseases that are poorly controlled; (2) Lactating or pregnant women; (3) Those with a history of other illness, including serious infectious disease; (4) Persons with incapacitated or limited ability to act; (5) Patients received radiotherapy or chemotherapy before surgery.

All patients were informed and consented to the collection of samples for research purposes, with signed informed consent forms for biospecimen acquisition. The research methods and specimen collection procedures involved in this study were submitted to and approved by the Ethics Committee of The First Affiliated Hospital of Sun Yat-sen University.

### Immunohistochemical staining

A total of 36 paraffin-embedded tissue from patients with HGSOC was stained by immunohistochemical (IHC) and Periodic Acid-Schiff (PAS) staining who received treatment at the First Affiliated Hospital of Sun Yat-sen University (Guangzhou, China). The formalin-fixed paraffin-embedded (FFPE) slides underwent deparaffinization in a graded series of xylene and ethanol. Epitopes were unmasked by immersing the slides in a boiling antigen retrieval solution for 5 minutes. Endogenous peroxidase activity was blocked with 3% hydrogen peroxide for 10 minutes and then incubated at room temperature for 25 minutes using goat serum blocking solution to eliminate non-specific staining. After incubation with mouse-derived anti-CD31 antibody (1:1000, CST, 3528S), the slides were placed at 4 degrees Celsius for 18 hours. Following PBS washing, an anti-mouse horseradish peroxidase-labeled secondary antibody (Vector Laboratories) was applied and incubated at 25°C for 45 minutes. After 2 min of with 0.05% 3′,3-diaminobenzidine tetrahydrochloride (DAB, ZSGB-BIO, ZLI-9017) staining, the PAS staining procedure was carried out in accordance with the ZSGB-BIO, BSBA-4080A manufacturer’s instructions. The slides were then dehydrated, mounted, and counterstained with hematoxylin.

### VM detection and distinguish

After the staining and neutral resin sealing process, the pathology slides were captured as electronic images at 40x magnification using a bright-field scanner. Subsequently, all channel-like structures were systematically examined based on the established criteria for typical vascular malformations. These criteria include the following: (1) VM vascular-like channels surrounded by tumor cells; (2) VM vessel channels staining PAS positive and CD31 negative (PAS+/CD31−), in contrast to endothelial vessel channels staining PAS positive and CD31 positive (PAS+/CD31+); (3) VM vascular-like channels containing erythrocytes (45). According to the results of IHC-PAS staining for VM, all samples of clinical specimens were categorized into two subgroups: VM (+) group meanings those with typical VM structures and VM (-) group meanings those without. The clinical characteristics of VM-positive and VM-negative groups are presented in **Supplementary Table S4**.

### Quantitative real-time PCR

In accordance with the manufacturer’s instructions, the total bulk RNA was extracted and purified through Trizol reagent (Invitrogen) and the SteadyPure Universal RNA Extraction Kit (ACCURATE BIOTECHNOLOGY (Human) CO., LTD, Changsha, China), and then it was reverse transcribed to cDNA through the Reverse Transcription Supermix (ACCURATE BIOTECHNOLOGY (Human) CO., LTD, Changsha, China, AG11706). The SYBR Green PCR Kit was utilized for conducting the qRT-PCR (ACCURATE BIOTECHNOLOGY (Human) CO., LTD, Changsha, China, AG11701) and in a Real-time fluorescence PCR instrument (Bio-Rad Laboratories, Inc, United States, Bio-Rad CFX Connect Real-Time PCR System 1855201). The expression of Glyceraldehyde 3-phosphate dehydrogenase (GAPDH) was set as an internal control to realize normalization. The Primers sequences in this study are presented in **Supplementary Table S5**. The comparative expression level was evaluated by 2^-ΔΔCt^ method.

### Statistical analysis

Using R software, statistical analyses were carried out. (version 4.4.1; http://www.Rproject.org). The categorical variables were compared using the Chi-square test. The Wilcoxon test was employed to compare the drug sensitivity and gene expression levels of groups. A *p*-value of less than 0.05 was taken into consideration as the criterion for statistical significance in this investigation.

## Result

### Interactions among vasculogenic mimicry-related genes in ovarian cancer

**Figure 1:**
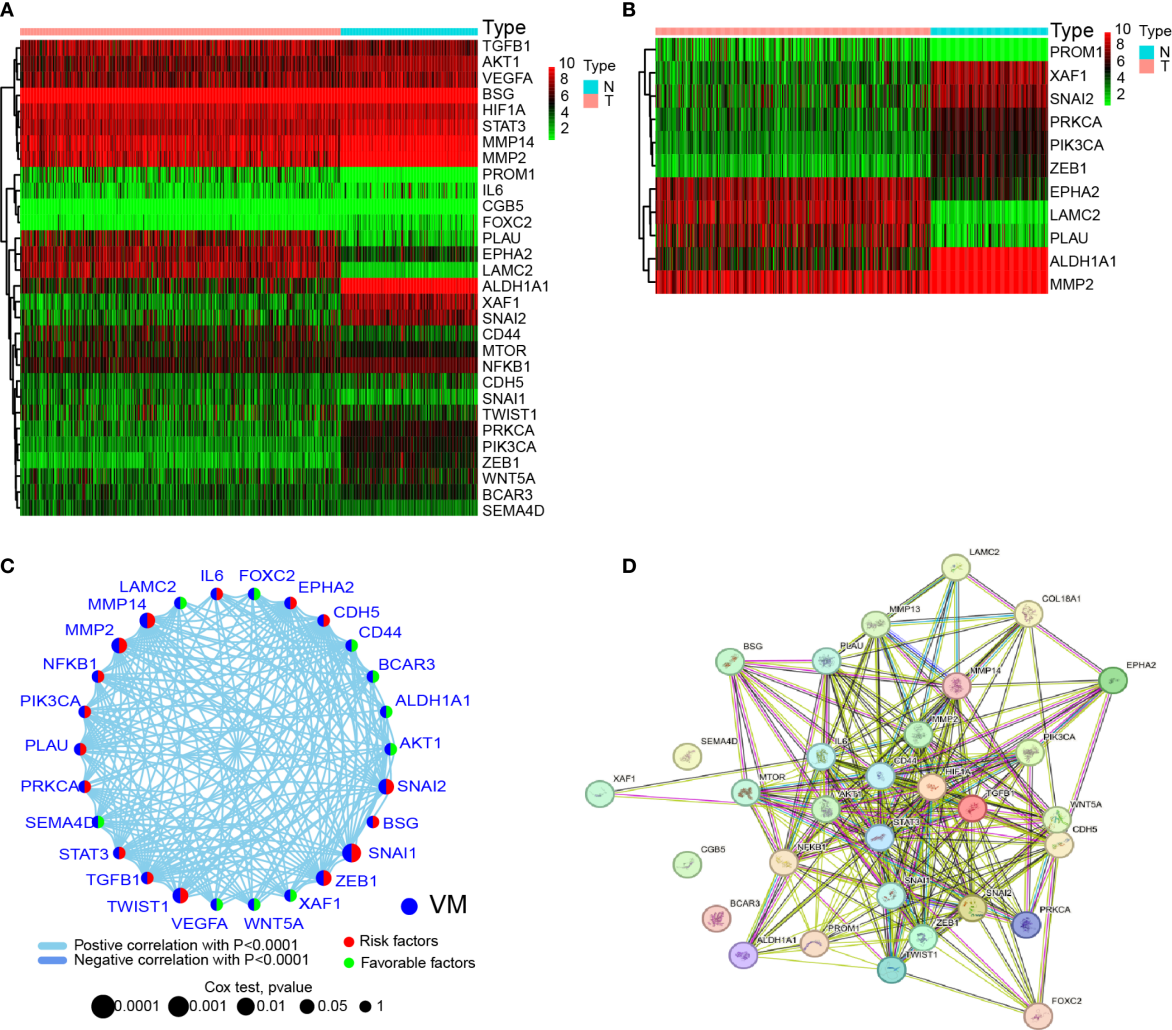
Expression and interactions of 30 VM-related genes. **(A)** Heatmap showing the differences in VMGs between GTEx normal samples (n=180) and TCGA OV tumor (n=429). Upregulation is represented by green, and downregulation by red. **(B)** A heatmap showing the differences in VMGs between GTEx normal samples (n=180) and TCGA OV tumors (n=429). Upregulation is represented by green, and downregulation by red. A threshold of *p* < 0.05 was chosen for the filtering. **(C)** VMGs’ mRNA expression-based correlation network. Positive and negative associations are respectively by the sky blue and dark blue lines. The red hemisphere represents risk factors, while the green hemisphere represents favorable factors. The size of the sphere depicts the strength of the hazard level. **(D)** VMGs’ network of protein-protein interactions (PPIs) (interaction score = 0.4). Both direct (physical) and indirect (functional) linkages are present in the exchanges.

To investigate whether VMGs exhibit differential expression between HGSOC and normal fallopian tube epithelial tissues and explore their carcinogenic roles at the genetic level, the TCGA dataset was utilized as the cancer tissue dataset. Since TCGA lacks large-scale sequencing data of normal fallopian tube epithelial tissues relevant to ovarian cancer, 180 normal fallopian tube epithelial tissue sequencing samples from GTEx were downloaded as controls. We first generated a heatmap of the data from the TCGA and GTEx databases to compare the expression levels of VM-related genes in HGSOC and normal fallopian tube tissues (**Figure 1A**). Subsequently, we analyzed significantly differentially expressed VMGs, and identified 11 VMGs, including PROM1, XAF1, SNAI2, PRKCA, ZEB1, EPHA2, LAMC2, PLAU, ALDH1A1, and MMP2, indicating aberrant transcription of VMGs in a substantial proportion of ovarian cancer lesions (**Figure 1B**).

Given that previous studies have shown that VM formation is driven by multiple genes and pathways, this study aimed to investigate potential interactions among VMGs. First, correlation analysis of 28 VMGs revealed complex interaction networks among these genes (**Figure 1C**). Additionally, since all studied VMGs encode proteins, PPIs network was constructed using public databases, revealing robust interactions among these genes at the protein level (**Figure 1D**). These findings suggest that VMGs may play critical roles in the initiation and progression of HGSOC, with intricate interaction relationships observed at both transcriptional and protein expression levels.

### Identification of subgroups associated with gene expression and prognosis

**Figure 2:**
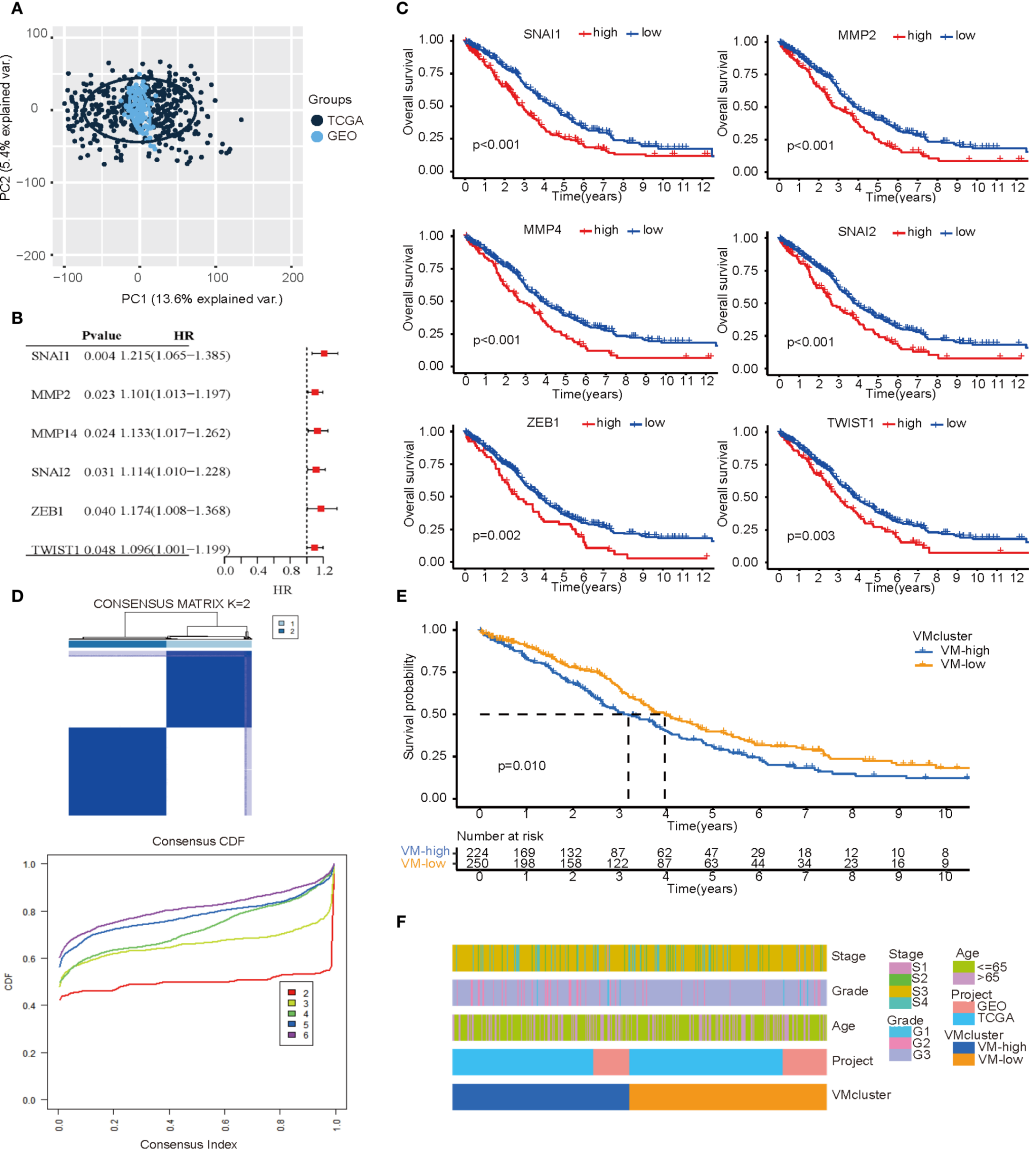
Subtypes based on VM-related genes expression. **(A)** PCA plot illustrating the combined TCGA and GEO data after batch effect removal. **(B)** A forest map of the most important VM-related genes for prognosis identified by univariate Cox regression analysis in OC patients. **(C)** Kaplan–Meier curves of important VM-related genes for prognosis. **(D)** Consensus clustering matrix (k=2) showing two clusters (VM-high = 224; VM-low = 250) based on the expression of important genes in 30 VMGs. **(E)** Overall survival showing a significant difference (*p* < 0.01) in the survival plot. **(F)** A heatmap showing the relationships among the patients’ clusters, clinicopathological characteristics, and data sources for ovarian cancer.

In order to expand the sample size, avoid overfitting to a single sequencing platform, and enhance generalizability across multiple platforms, this study integrated TCGA and GEO datasets after batch effect removal to establish the “primary dataset” with PCA confirming post-merger consistency (**Figure 2A**). Preliminary experiments demonstrated that direct consensus clustering using all 33 VMGs failed to achieve precise molecular subtyping. Therefore, we selected VMGs significantly associated with prognosis as determinants for subtyping. Survival analysis of 33 VMGs in HGSOC patients identified 6 VMGs (SNAI1, SNAI2, MMP2, MMP14, ZEB1, and TWIST1) with prognostic relevance, all exhibiting hazard ratios (HR) > 1, suggesting that their high expression correlates with poor outcomes in ovarian cancer (**Figure 2B**). Kaplan-Meier (KM) curves confirmed significant survival differences between high- and low-expression groups (**Figure 2C**). Consensus clustering based on expression profiles of VM-related prognostic genes was performed to identify subtypes. At k=2 (number of clusters), the cumulative distribution function (CDF) curve displayed optimal smoothness, indicating maximal intra-group homogeneity and inter-group heterogeneity. Consequently, the primary dataset was stratified into two subgroups: VM-high (n= 224) and VM-low (n= 250) (**Figure 2D**). Notably, the VM-low subgroup exhibited significantly better overall survival compared to the VM-high subgroup (**Figure 2E**). Statistical validation confirmed no significant differences in sequencing data sources or distributions of key clinical characteristics between subgroups, as visualized by heatmap (**Figure 2F**).

### Enrichment analysis of DEGs and construction of prognostic model

**Figure 3:**
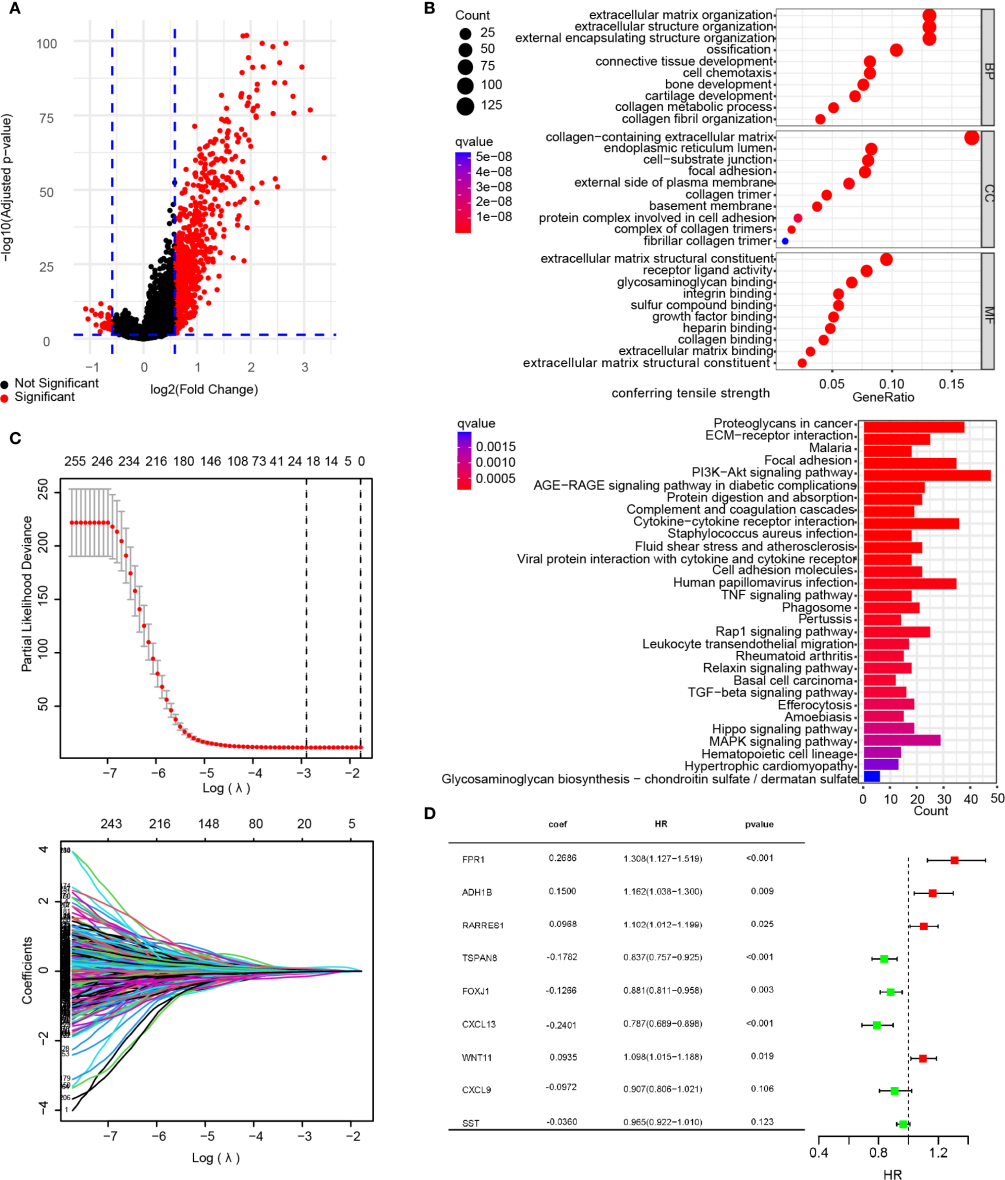
Construction of a risk signature. **(A)** Volcano map showing DEGs between the VMG clusters **(A)**. DEGs were sifted using the subsequent standards: log_2_ fold alteration (log_2_ FC) =0.585, and a false discovery rate (FDR) < 0.05. **(B)** The bar plots display the results of GO and KEGG enrichment analysis for the DEGs. **(C)** A total of 260 Overall Survival-related genes were identified by univariate Cox regression analysis and subjected to LASSO regression, using cross-validation to fine-tune the LASSO regression’s parameter selection. **(D)** Forest plot of the important genes and coefficients in the gene model by multivariate Cox regression analysis.

In the primary dataset, parameters were set as log_2_ FC = 0.585 and FDR<0.05 to screen DEGs between the subgroups, identifying 758 statistically significant DEGs. Among these, 728 genes were significantly upregulated in the VM-high subgroup compared to the VM-low subgroup, while 30 genes were downregulated. A volcano plot illustrates the distribution of DEGs between subgroups (**Figure 3A**). GO and KEGG analyses were performed to explore the biological functions and pathways associated with the DEGs. GO analysis revealed that the top five enriched biological processes (BP) terms were “extracellular matrix organization”, “extracellular structure organization”, “external encapsulating structure organization”, “ossification” and “connective tissue development”. The top five enriched cellular component (CC) terms were “collagen−containing extracellular matrix”, “endoplasmic reticulum lumen”, “cell−substrate junction”, “focal adhesion” and “external side of plasma membrane”. The top five enriched molecular function (MF) terms were “extracellular matrix structural constituent”, “receptor ligand activity”, “glycosaminoglycan binding”, “integrin binding” and “sulfur compound binding”. These findings suggest alterations in extracellular matrix metabolism and intercellular junction states between subgroups. The KEGG analysis showed that DEGs were mainly involved in proteoglycans in the cancer signaling pathway, the ECM−receptor interaction signaling pathway, the Malaria signaling pathway, the focal adhesion signaling pathway, the PI3K−Akt signaling pathway signaling pathway, the AGE−RAGE signaling pathway in the diabetic complications signaling pathway, The protein digestion and absorption signaling pathway, the complement and coagulation cascades signaling pathway, the cytokine−cytokine receptor interaction signaling pathway, and the staphylococcus aureus infection signaling pathway (**Figure 3B**). These results indicate that these pathways are modulated following VM formation and may contribute to prognostic disparities.

Furthermore, to explore the prognostic relevance of VM-related DEGs in ovarian cancer, univariate Cox regression analysis was first performed on the DEGs, identifying 260 prognosis-associated DEGs (**Supplementary Table S6**). The primary dataset was randomly divided into a training set (80% cases) and an internal validation set (remaining 20% cases) using R functions. LASSO regression analysis was applied to refine the 260 prognostic DEGs, ultimately selecting an optimal 9-gene subset (FPR1, ADH1B, PARRES1, TSPAN8, FOXJ1, CXCL13, WNT11, CXCL9, and SST) with superior prognostic performance (**Figure 3C**). Multivariate Cox analysis was then conducted to calculate prognostic coefficients (β) for the selected genes, presented as a forest plot. The results indicated that FPR1, ADH1B, RARRES1, and WNT11 expression levels correlated with higher risk scores, whereas TSPAN8, FOXJ1, CXCL13, CXCL9, and SST were associated with lower risk scores. These genes were defined as the VMRPI (**Figure 3D**). Based on these findings, a prognostic model comprising the 9 genes was established, with the risk score calculated as: Risk Score = FPR1×0.2686 + ADH1B×0.1500 + RARRES1×0.0968 + TSPAN8×(-0.1782) + WNT11×0.0935 + FOXJ1×(-0.1266) + CXCL13×(-0.2401) + CXCL9×(-0.0972) +SST×(-0.0360), where gene names represent their TPM values in sequencing data.

### Validation of the prognostic model and gene expression level

**Figure 4:**
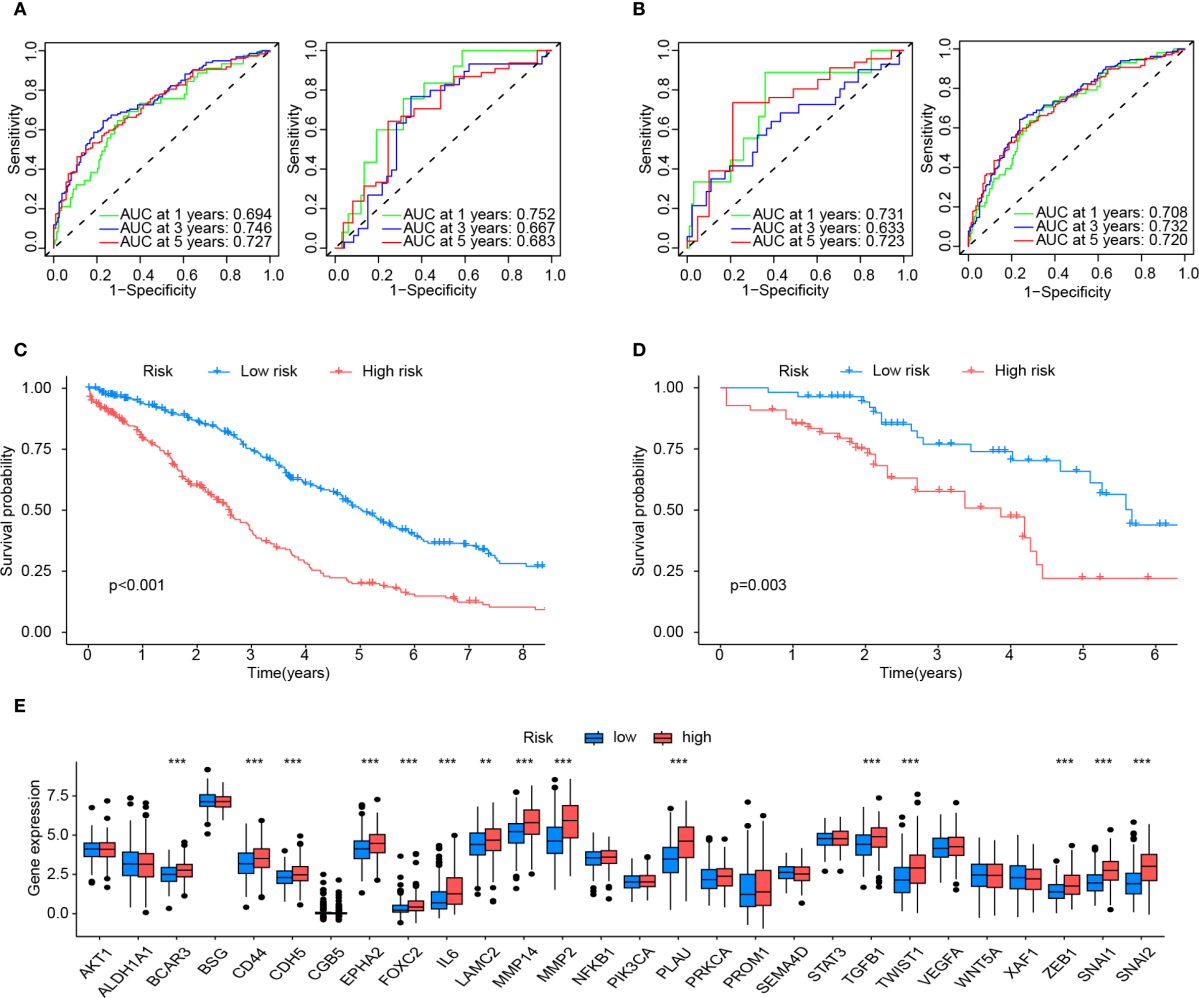
Validation and assessment of the risk model. **(A)** The predictive effectiveness of the risk score in the training set (left) and test sets (right) is shown by the time-dependent receiver operating characteristic (ROC) curve and the area under curve (AUC) analyses. **(B)** Shows the predictive effectiveness of the risk score in all sets (left) and in the external validation set (right) using time-dependent ROC curves and AUC analyses. **(C)** Kaplan-Meier curves for the Overall survival (OS) of VMRPI-High and VMRPI-Low patients in the total cohort. **(D)** Kaplan-Meier curves for the OS of VMRPI-High and VMRPI-Low patients in the external validation cohort. **(E)** In TCGA cohort, the expression levels of VM-related genes were compared between the VMRPI-High and VMRPI-Low subgroups. In results’ figures, ns means not significant, ** means *p* < 0.01, and *** means *p*< 0.001.

The prognostic efficacy of the model was evaluated using the AUC values from ROC analysis, demonstrating satisfactory predictive performance. In the training cohort, the 1-, 3-, and 5-year AUC values were 0.694, 0.746, and 0.727, respectively, while the internal validation cohort yielded corresponding values of 0.752, 0.667, and 0.663 for the same intervals (**Figure 4A**). To further validate the model’s generalizability and mitigate overfitting to the primary dataset, the external validation cohort demonstrated robust concordance with the primary dataset, yielding 1-, 3-, and 5-year AUC values of 0.731, 0.633, and 0.723, respectively, compared to the primary dataset’s corresponding values of 0.708, 0.732, and 0.720 (**Figure 4B**).

Samples from both the primary dataset and the external validation cohort were stratified into high-risk and low-risk groups based on the median risk score of each cohort. Survival analysis revealed statistically significant disparities in survival outcomes between risk groups across both datasets, with all *p*-values <0.05 (**Figures 4C, D**). Furthermore, intergroup differential gene analysis revealed significant differences in the expression of VMGs, including BCAR3, CD44, CDH5, EPHA2, FOXC2, IL6, LAMC2, MMP14, MMP2, PLAU, TGFB1, TWIST1, ZEB1, SNAI1, and SNAI2 (**Figure 4E**). These results comprehensively underscore the robust prognostic significance of the risk score.

### Evaluation of the clinical features of risk subgroups and calculation of the nomogram

**Figure 5:**
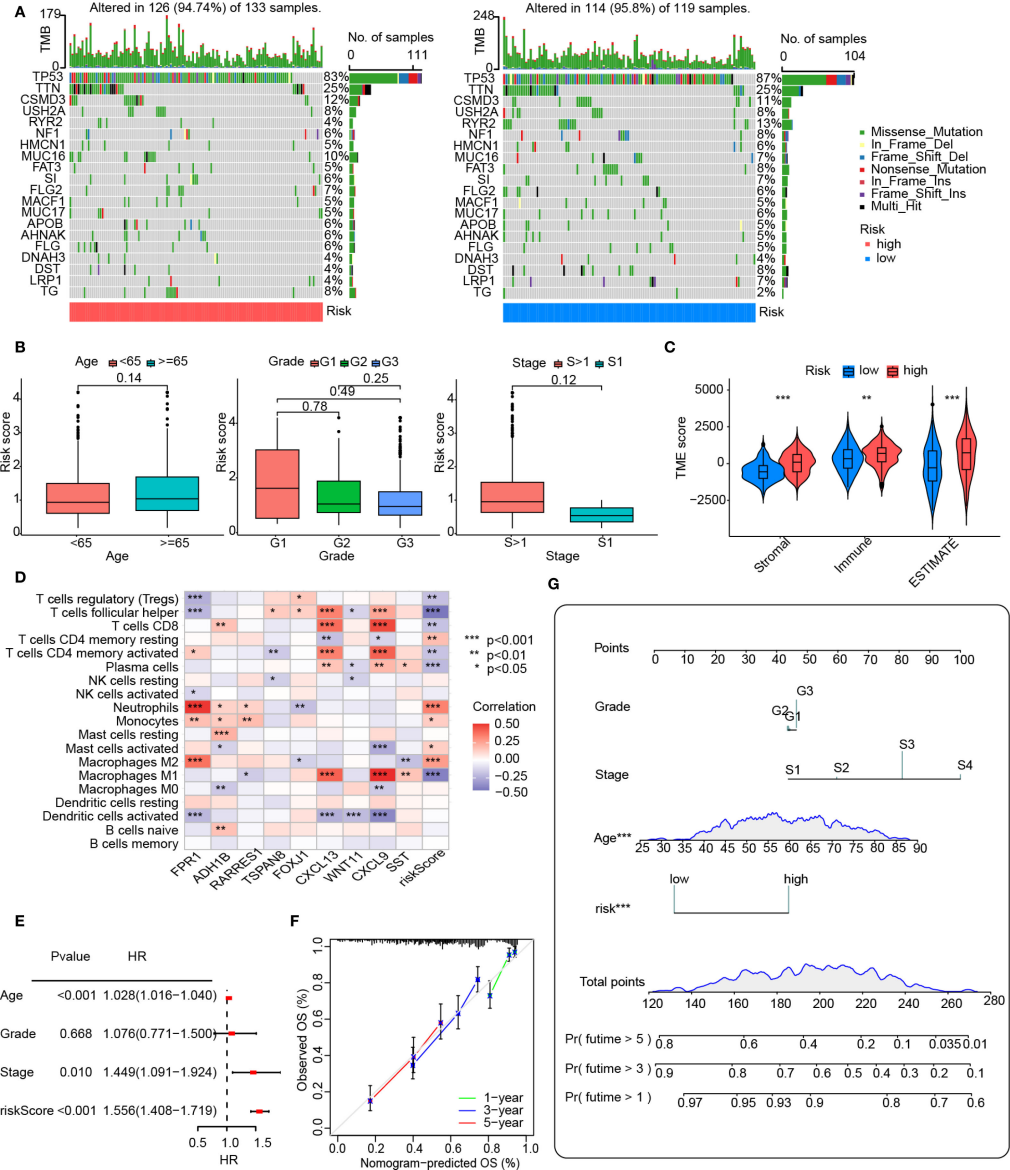
Clinical analysis and nomogram construction. **(A)** An oncoplot showing the frequency of mutations in the top 20 mutated genes in the high- and low-risk groups. **(B)** Boxplot showing the age, grade, and FIGO stage for the groups at high and low risks. The numbers above the horizontal lines represent the p-values from the statistical tests. **(C)** Distribution of immunological, stromal, and ESTIMATE scores based on VMRPI. **(D)** Spearman’s correlation between VMRPI genes and immune cells in the TCGA-OV dataset was determined using the TIMER database. **(E)** A forest map of prognostic clinical indices using a nomograph. **(F)** Nomogram’s calibration curves. The nomogram-predicted likelihood of invasive adenocarcinoma is represented by the x-axis, whereas the actual probability is represented by the y-axis. **(G)** Nomogram using tumor grade, Figo staging system, age, and VMRPI. In results’ figures, ns means not significant, * means *p* < 0.05, ** means *p* < 0.01, and *** means *p* < 0.001.

Stratification based on risk scores was utilized to analyze tumor mutational profiles and clinical prognostic factors. The tumor mutational profile analysis incorporated mutation data from the TCGA database. The results revealed that the top five mutated genes in both high-risk and low-risk groups were TP53, TTN, CSMD3, USH2A, and RYR2, indicating that risk score stratification does not significantly alter the predominant mutational landscape of tumors (**Figure 5A**). To investigate potential associations between risk score stratification and clinical prognostic factors (including age, tumor grade, and clinical stage), statistical analyses were performed on clinical subgroups. No significant differences in risk scores were observed across age groups, tumor differentiation status, or tumor stages, suggesting that the risk score functions as a prognostic factor independent of these clinical variables (**Figure 5B**).

Furthermore, given the critical role of immune status in tumorigenesis, recurrence, and prognosis, we explored correlations between risk scores and immune profiles by analyzing immune-stromal scores and immune cell infiltration. Immune-stromal scoring stratified by risk groups demonstrated significantly higher immune scores, stromal scores, and ESTIMATE scores in the high-risk group compared to the low-risk group (**Figure 5C**). Additionally, the heatmap analysis, however, revealed significant associations between VMRPI and risk scores and infiltration levels of various immune cell subtypes. These notably included neutrophils, monocytes, activated mast cells, M2 macrophages, resting memory CD4+ T cells, follicular helper T cells, plasma cells, regulatory T cells, M1 macrophages, CD8+ T cells, and activated memory CD4+ T lymphocytes (**Figure 5D**). Furthermore, ICG profiling demonstrated differential expression patterns of specific ICGs between high- and low-risk groups, unveiling a complex immune checkpoint landscape (**Supplementary Figure S1**).

To integrate the prognostic model with clinical factors for quantitative survival probability prediction, a nomogram was constructed and visualized. Univariate Cox analysis of the primary dataset identified age, clinical stage, and the prognostic model as significant predictors (**Figure 5E**). Although tumor grade showed no statistical significance due to limited low-grade samples, it was retained in nomogram construction based on established clinical guidelines, pathological consensus, and prior evidence supporting its prognostic relevance. Multivariate Cox analysis successfully integrated clinical features with risk scores to generate a nomogram for quantitative prognosis assessment and calibration curves confirmed robust predictive accuracy at 3-year and 5-year intervals (**Figures 5F, G**).

### Prediction of therapeutic response to drugs based on the risk score

**Figure 6:**
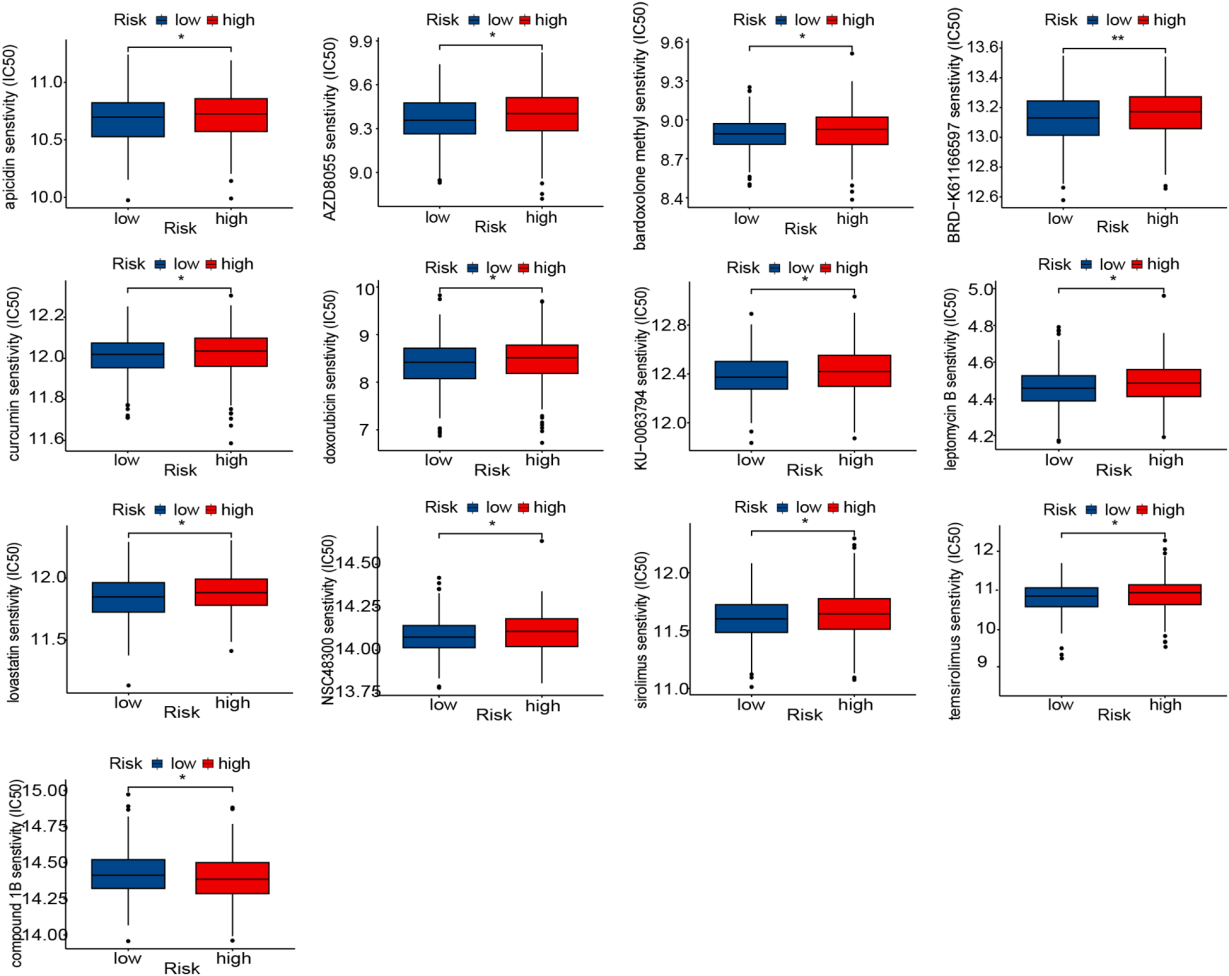
Prediction of therapy response to drugs. Boxplot of therapeutic response to drugs in the high- and low-risk groups. In the figures of results, * means *p* < 0.05, and ** means *p* < 0.01.

As a critical prognostic factor in molecular subtypes, the exploration of VMRPI in clinical therapeutic strategies holds significant importance. Therefore, this study conducted drug sensitivity analysis based on risk score stratification utilizing the CTRP and GDSC databases. Based on risk score-based stratification, the sensitivity scores of various drugs indicated that drugs, such as BRD-K61166597, apicidin, AZD8055, bardoxolone methyl, curcumin, doxorubicin, KU-0063794, lovastatin, NSC48300, leptomycin B, sirolimus, and temsirolimus, exhibit higher IC_50_ values within the high-risk group. Conversely, compound 1B displayed lower IC_50_ values (**Figure 6**). These results suggest that partial differences in drug sensitivities exist among subgroups stratified by risk scores, and that for patients with high-risk scores, drugs with better responsiveness can be administered while avoiding those with poor responsiveness.

### Experimental expression validation of cluster genes and VMRPI

**Figure 7:**
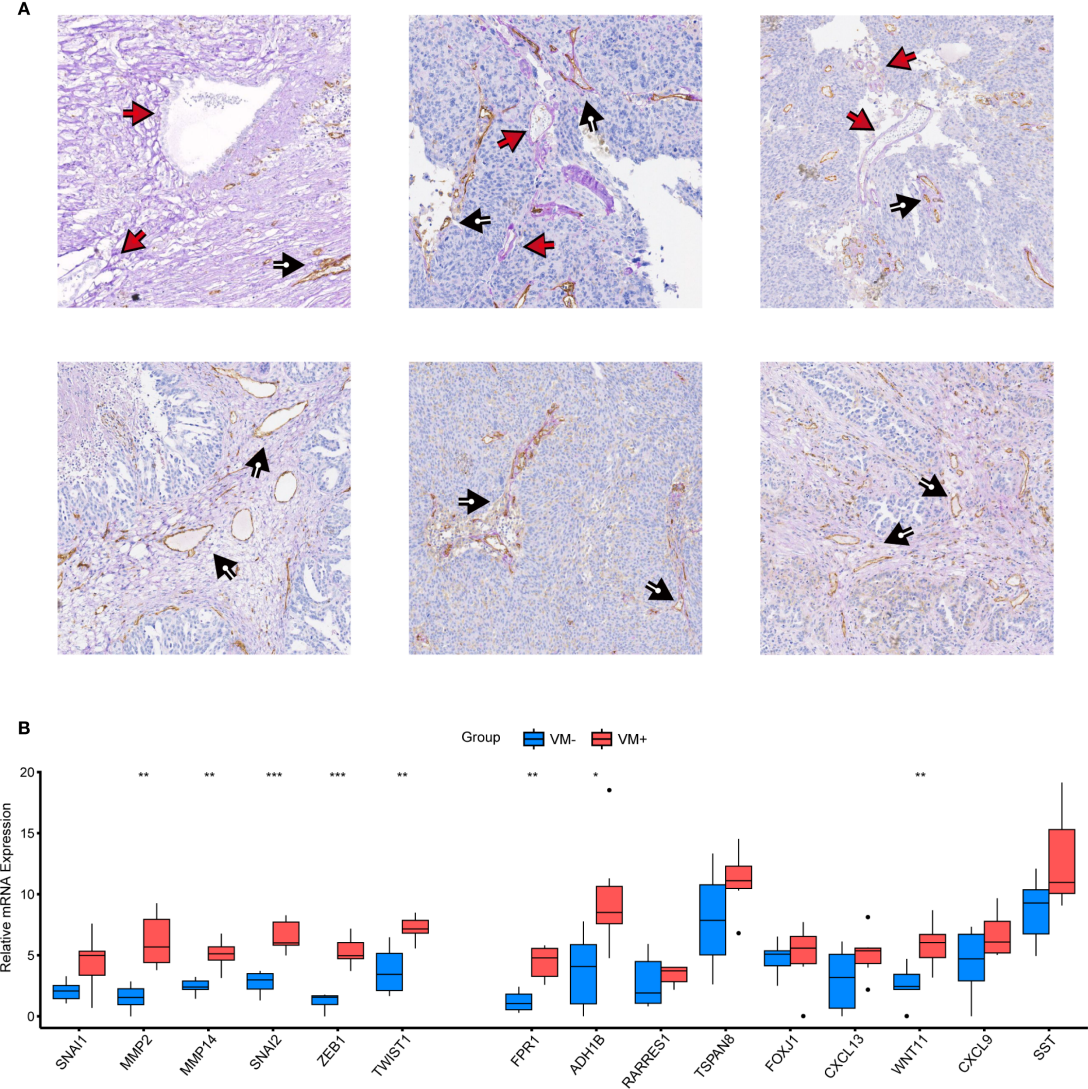
Experimental validation of VM genes and VMRPIs. **(A)** Vascular structures in HGSOC. The black arrows denote CD31+/PAS+ endothelial angiogenic structures, whereas the red arrows denote CD31−/PAS+ vasculogenic mimicry structures. **(B)** qRT–PCR was performed to validate the differences in VM cluster genes and VMRPIs between the two groups (VM+, n=6; VM-, n=30). The two groups were classified based on IHC-PAS staining results: samples with typical VM structures observed under microscopy were designated as the VM+ group, while those without obvious VM structures were classified as the VM- group. The data are presented as the mean ± SD. The statistical study was conducted using the Wilcoxon test. In the figures of results, nothing means not significant, * means *p* < 0.05, ** means *p* < 0.01, and *** means *p* < 0.001.

To further investigate whether VM structures correspond to VMRPIs in biological samples, we further explored using clinical specimens. Paraffin-embedded specimens and paired fresh frozen tissues from 36 cases of primary HGSOC in our center were used for IHC-PAS staining. After careful examination, 6 cases exhibited typical VM structures— specifically, basement membrane-lined, tumor cell-encircled luminal structures containing erythrocytes— with negative CD31 staining, consistent with the proportion of VM-positive patients reported in prior ovarian cancer studies (**Figure 7A**). Subsequently, 36 patients were categorized into VM-positive and VM-negative groups based on IHC-PAS results, with three representative images selected from each group for presentation. Corresponding fresh frozen tissues were then subjected to qRT-PCR to detect the expression of VMGs and VMRPIs. The results revealed statistically significant differences in the expression of the representative VMGs, including MMP2, MMP14, SNAI2, ZEB1, and TWIST1, between the VM+ and VM- groups. Moreover, FPR1, ADH1B, and WNT11, identified as VMRPIs, also exhibited statistically significant gene expression differences and demonstrated a relationship with VM that aligned with the prognostic risk analysis (**Figure 7B**). These findings further clarify the association between upregulated FPR1, ADH1B, and WNT11 expression and VM structures. The clinical characteristics of the VM-positive and VM-negative groups, including age, grade, and stage, are presented in **Supplementary Table S4**.

## Discussion

Clinically, it is well recognized that patients with HGSOC, which is the most common pathological subtype and accounts for the majority of both incidence and mortality in OC, and who have comparable age, identical pathological types, and similar differentiation degrees, may exhibit markedly divergent prognostic outcomes (46). This discrepancy primarily stems from the failure of traditional clinical prognostic classifications to incorporate molecular heterogeneity among such patients into consideration (47, 48). Since its initial discovery in melanoma research in 1999, VM has been validated across multiple solid tumors and consistently correlates with inferior prognostic outcomes (16, 17). Despite the robust prognostic relevance of VM and incremental elucidation of its mechanistic foundations and biological implications, no studies to date have exploited this prognostic determinant to develop molecular subclassification frameworks or predictive models for HGSOC survival assessment (49, 50). Therefore, this study aims to stratify ovarian cancer patients molecularly based on prognosis-related genes associated with VM, a prognostic factor in ovarian cancer. Furthermore, using DEGs, we constructed a prognostic model via the Lasso-Cox algorithm, which suggests associations with immune infiltration and drug sensitivity. Experimental validation also confirmed that some VMRPIs are highly expressed in the VM-positive group.

Concerning the molecular underpinnings of VM formation in ovarian cancer, beyond the well-characterized contributions of key pathways - including the VE-cadherin/EphA2/MMPs axis, hypoxia-driven HIF-1A signaling, and EMT - associated regulators - genes such as ALDH1A1, BCAR3, and CGB5 have been identified as critical mediators in this process (25, 26, 28). Therefore, based on literature review, we selected the above-mentioned genes as the VM-related gene set. Compared with normal fallopian tube epithelium, 11 VMGs were highly expressed in HGSOC tissues. However, preliminary experiments indicated that these 11 VMGs could not stratify ovarian cancer patients by prognosis, suggesting that they might be involved in the aberrant expression patterns of HGSOC without exerting significant prognostic impact. Consequently, we performed prognostic analysis of the VMGs and identified 6 VMGs that effectively stratified patients into distinct prognostic subgroups. Interestingly, functional enrichment analysis of the DEGs, identified between prognostic subgroups, revealed that the involved pathways and cellular processes were primarily associated with EMT and ECM remodeling. This is consistent with previous findings that VM formation in ovarian cancer follows a stepwise biological process: initially, stemness-acquired tumor cells metabolize the matrix and connect with vasculature, followed by EMT-mediated functional acquisition, ultimately leading to the formation of an interconnected perfusion network (22, 23, 28).

In the evaluation of model efficacy, promising results were achieved not only in the internal validation dataset but also in the external validation cohort, indicating strong generalizability of the model. Moreover, the risk score showed no significant correlation with established clinical prognostic factors such as age, grade, and clinical stage, confirming that the prognostic discrimination capability of the model is independent. Given that multivariate Cox regression identified the risk score as an independent prognostic factor, we combined conventional clinical parameters with this risk scoring system to develop a nomogram for quantitatively predicting survival probabilities, thereby enhancing the accuracy of clinical prognostic assessment. However, there remains room for improvement in model performance. This includes: (1) employing more refined stratification strategies, though acquiring large-sample real-world data with high precision remains challenging (51); (2) utilizing more advanced machine learning or deep learning methods, which may, however, suffer from poor biological interpretability (52, 53); and (3) incorporating multi-omics data to construct a comprehensive prognostic model, albeit at the potential cost of increased experimental expenses and complexity (54). In conclusion, the rigorous feature selection via LASSO-Cox regression supports the feasibility of constructing a prognostic model based on VM-related stratification.

Following rigorous screening and computational optimization, a multivariate risk scoring model was ultimately established through integration of expression profiles and regression coefficients from nine core genes: FPR1, ADH1B, RARRES1, TSPAN8, FOXJ1, CXCL13, WNT11, CXCL9, and SST. Accumulating evidence from mechanistic and clinical studies has implicated these model-incorporated genes in tumor biological processes, as documented in existing oncology literature. C-X-C motif chemokine ligand 13 (CXCL13), known as a fundamental regulator of B-cell recruitment and organization, can coordinate the development of tertiary lymphoid structures (55). Its expression level is associated with long-term survival and can enhance the effectiveness of PD-1 checkpoint blockade in HGSOC (56, 57). Formyl peptide receptor 1 (FPR1) is not only in proinflammatory and antibacterial host responses but also involved in cell chemotaxis, proliferation, and tumor progression (58–60). Activated FPR may contribute to these processes and has been identified as a potential biomarker and treatment target for aggressive epithelial ovarian cancer (EOC) (61, 62). Tetraspanin 8 (TSPAN8) is a member of the tetraspanin family, implicated in various human cancers through its role in regulating intercellular interactions and cell motility (63, 64). It is a potential therapeutic target for the inhibition of invasion and metastasis in OCs (65–67). Forkhead box J1 (FOXJ1) is a 3-exon transcription factor and a master regulator of motile ciliogenesis (68–71). It is expressed in various tissues such as the respiratory tract, brain, and reproductive tract, where it plays a pivotal role in regulating transcriptional programs governing motile cilia assembly. This activity showcases its diverse implications for cancer biology and prognosis. High FOXJ1 expression is correlated with better tumor differentiation and favorable prognosis in various cancers such as gastric cancer, ependymomas, choroid plexus tumors, and ovarian cancer (72–74). Alcohol dehydrogenase class I beta polypeptide (ADH1B) is pivotal for alcohol metabolism and is implicated in tumorigenesis, particularly, in esophageal squamous cell and colorectal cancers (75–77). High FABP4 and ADH1B expressions in high-grade serous ovarian cancer suggest an increased risk of residual disease, possibly guiding neoadjuvant chemotherapy candidacy (78). Wnt family member 11 (WNT11) is a noncanonical Wnt protein that regulates cell movement and organ formation through specific receptors and signaling pathways (79–84). It plays a dual role by promoting migration in certain cancers including breast cancer, colon cancer, and leukemia, while suppressing cell migration in hepatocellular carcinoma (85, 86). Additionally, WNT11 influences cell adhesion and migration by modulating the expression of E-cadherin and integrin subunits (85, 87). Retinoic acid receptor responder 1 (RARRES1, also known as tazarotene-induced gene 1, TIG1) is upregulated by tazarotene in skin culture and resembles CD38 and is frequently silenced in cancers due to promoter hypermethylation. It shows potential as a tumor suppressor in prostate and endometrial cancers (88–90). Notably, RARRES1 plays a crucial role in promoting tumor growth and invasion in IBC through Axl, indicating its promise as a therapeutic target for IBC patients (91). C-X-C motif chemokine ligand 9 (CXCL9), in conjunction with CXCL10 and CXCL11, boosts T-cell infiltration in OC, and is associated with improved survival rates (92). Research indicates that CXCL9 and its chemokine counterparts are indicators of an inflammation-rich subtype of ovarian cancer (93, 94). Recent evidence highlighted the predictive value of CXCL9 for positive outcomes and favorable responses to anti-PD-1 therapy in cancer patients (95). Somatostatin (SST) is cyclic peptide that inhibits hormone secretion and suppresses immune functions (96–99). Its signaling pathway also regulates tumor characteristics such as angiogenesis, cell migration, and growth factors, promoting tumor neovascularization and cell growth (96, 100–102). For these risk genes, there is an urgent need for further mechanistic investigations to elucidate their role in mediating VM during ovarian carcinogenesis.

Immunological evaluation results indicated that risk score stratification correlated with the distribution of specific immune cell subsets and ICGs expression, while immune-stromal scoring revealed elevated scores in the high-risk group. This phenomenon may be attributed to potential increases in VM structures within high-risk populations. Under such circumstances, alterations in the tumor microenvironment could trigger immune cell redistribution, elevated concentrations of immune factors, and enhanced infiltration status. However, in high-risk group patients, the reduction of tumor-killing immune cells along with suppression of specific immune checkpoints ultimately results in a persistently suboptimal immune status. Immunological subtypes are also closely associated with prognosis and can serve as predictors of drug responsiveness (103). For instance, recent studies have demonstrated that subtypes defined based on responsiveness to immune checkpoint inhibitors can be used to construct prognostic models with excellent performance (104). Therefore, we also predicted drug responsiveness in patients with different risk profiles. The results demonstrated that patients in the high-risk prognostic score group may exhibit suboptimal therapeutic responses to histone deacetylase 7/8 inhibitors (Apicidin), mTOR inhibitor-1 (AZD8055), bardoxolone methyl, histone deacetylase 1/2 inhibitors (BRD−K61166597), curcumin, doxorubicin, mTOR inhibitor-2 (KU−0063794), nuclear export inhibitors (LeptomycinB), lovastatin, small-molecule threonine endopeptidase inhibitors (NSC48300), sirolimus, and temsirolimus, while showing enhanced responsiveness to Compound 1B. Compound 1B, initially characterized as a dynein-targeting agent, was later identified as a member of the phosphodiesterase inhibitor family (105). While it exhibits selective cytotoxicity in cancer cells by promoting PDE3A-SLFN12 interaction and inducing cell death—particularly in KRAS-mutant cancers—its role in ovarian cancer remains poorly studied (106, 107). Thus, model-based drug sensitivity prediction is essential not only to guide the selection of conventional chemotherapeutics but also to highlight promising agents warranting further investigation.

To further validate the relationship between VM structure and VMRPI, we conducted experiments using samples from our center. Results from IHC-PAS staining on paraffin-embedded sections showed that VM structures were present in 6 out of 36 samples, which is consistent with previously reported rates of VM occurrence in HGSOC patients (50). qRT-PCR analysis of matched frozen samples revealed that VM-associated genes—MMP2, MMP14, SNAI2, ZEB1, and TWIST1—exhibited significant expression differences between VM-positive and VM-negative groups. Furthermore, VMRPI markers including FPR1, ADH1B, and WNT11 showed elevated expression in VM-positive patients compared to VM-negative ones. These findings confirm the consistency between bioinformatic predictions and pathological observations, thereby reinforcing the association between VM and VMRPI and suggesting their potential role in HGSOC pathogenesis as well as highlighting avenues for further mechanistic investigation.

However, this investigation has limitations: First, the observation of higher immune scores in high-risk subgroups and specific patterns of immune checkpoint necessitate further mechanistic exploration to elucidate the underlying causes and their relationship with poor prognosis. Second, multi-center retrospective studies with larger sample sizes are required to integrate the risk scoring model into existing clinical staging systems or molecular classification-based prognostic frameworks, complemented by prospective cohort studies to validate its clinical utility in practical settings.

## Conclusion

**Figure 8:**
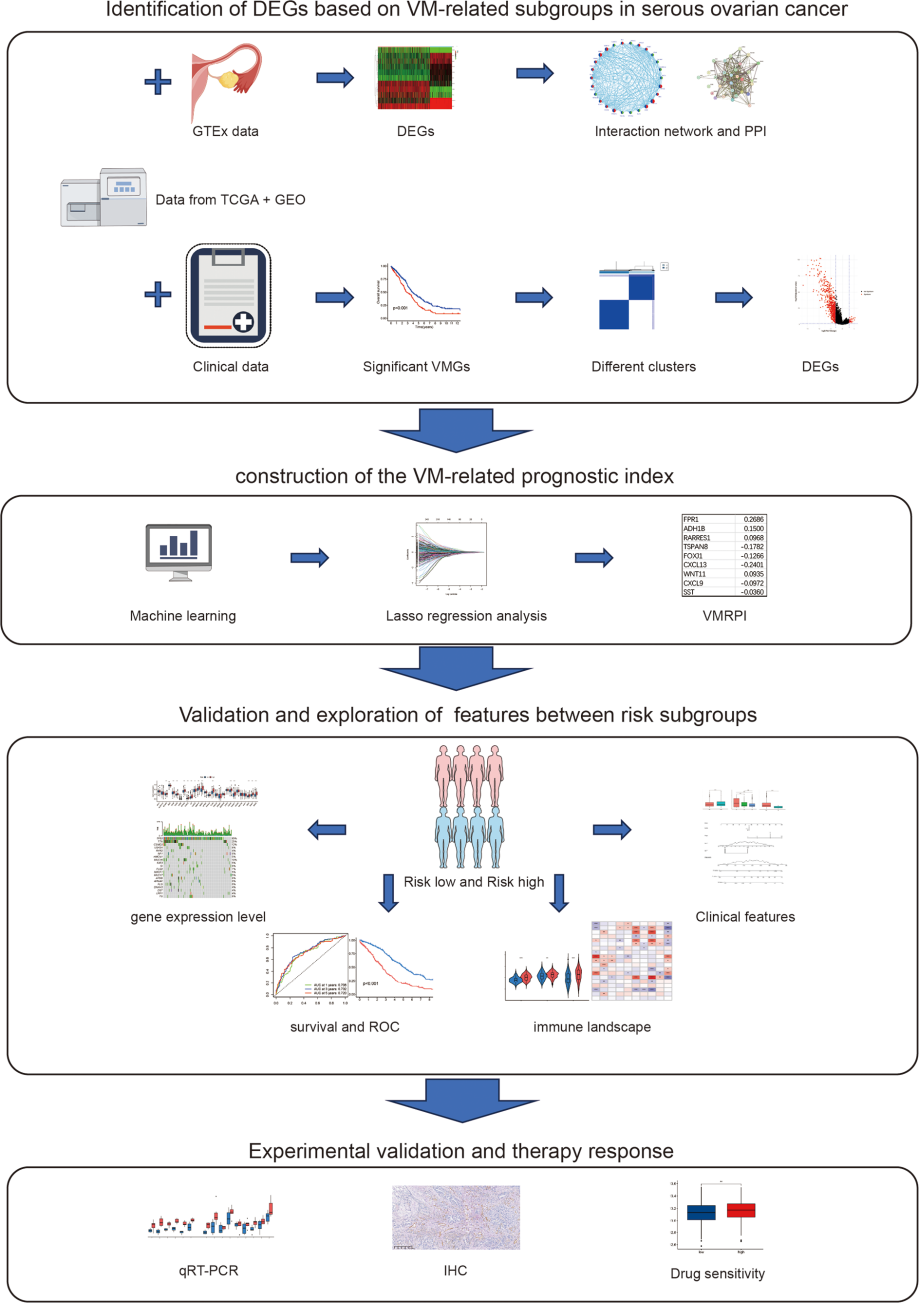
The study research process.

This study established a prognostic model and nomogram incorporating VM-related prognostic index, including FPR1, ADH1B, RARRES1, TSPAN8, FOXJ1, CXCL13, WNT11, CXCL9, and SST, which demonstrated favorable performance in prognostic evaluation and risk stratification of HGSOC patients. Furthermore, our findings revealed that risk stratification exhibited correlations with the immune microenvironment and drug sensitivity, thereby providing additional clinical applicability and research implications. Finally, experimental validation in clinical specimens confirmed the presence of VM structures and intergroup differential expression of associated genes (**Figure 8**).

## Data Availability

The datasets presented in this study can be found in online repositories. The names of the repository/repositories and accession number(s) can be found below: <b> https://portal.gdc.cancer.gov, serous ovarian cancer https://www.ncbi.nlm.nih.gov/geo/, GSE51088 https://www.ncbi.nlm.nih.gov/geo/, GSE17260 https://www.gtexportal.org/, fallopian tube RNA sequencing.
