# Supplementary material for: Establishment and validation of a prognostic model based on vasculogenic mimicry-related gene clustering in ovarian cancer
Source: Front Oncol. 2025 Sep 25;15:1575694. doi: 10.3389/fonc.2025.1575694 (PMC12507593; doi:10.3389/fonc.2025.1575694)
Supplement: Supplementary file 1 [file DataSheet1.docx]

Supplementary Material

**Establishment and validation of a prognostic model based on vasculogenic mimicry-related gene clustering in ovarian cancer**

**Supplementary Table**

| **Table S1. the Summary Table of Patient Cohorts** | | | |
| --- | --- | --- | --- |
| **Variables** | **the primary dataset** | | **the external validation dataset** |
| Data source | TCGA-HGSOC | GSE51088 | GSE17260 |
| Usable data | 299 | 94 | 110 |
| Age | 59.53±11.738 | 59.39±12.347 | NA |
| Grade |  |  |  |
| G1 | 0 | 0 | 0 |
| G2 | 0 | 0 | 0 |
| G3 | 299 | 94 | 110 |
| Stage |  |  |  |
| FIGO I | 10 | 3 | 0 |
| FIGO II | 16 | 3 | 0 |
| FIGO III | 237 | 78 | 33 |
| FIGO IV | 36 | 10 | 11 |

| **Table S2. Vasculogenic Mimicry-related Genes List** | | |
| --- | --- | --- |
| **No.** | **Gene symbol** | **Descriptions** |
| 1 | AKT1 | AKT serine/threonine kinase 1 |
| 2 | ALDH1A1 | aldehyde dehydrogenase 1 family member A1 |
| 3 | BCAR3 | BCAR3 adaptor protein, NSP family member |
| 4 | BSG | basigin (Ok blood group) |
| 5 | CD44 | CD44 molecule (IN blood group) |
| 6 | CDH5 | cadherin 5 |
| 7 | CGB5 | chorionic gonadotropin subunit beta 5 |
| 8 | EPHA2 | EPH receptor A2 |
| 9 | FOXC2 | forkhead box C2 |
| 10 | HIF1A | hypoxia inducible factor 1 subunit alpha |
| 11 | IL6 | interleukin 6 |
| 12 | LAMC2 | laminin subunit gamma 2 |
| 13 | MIR200A | microRNA 200a |
| 14 | MIR27B | microRNA 27b |
| 15 | MIR765 | microRNA 765 |
| 16 | MMP14 | matrix metallopeptidase 14 |
| 17 | MMP2 | matrix metallopeptidase 2 |
| 18 | MTOR | mechanistic target of rapamycin kinase |
| 19 | NFKB1 | nuclear factor kappa B subunit 1 |
| 20 | PIK3CA | phosphatidylinositol-4,5-bisphosphate 3-kinase catalytic subunit alpha |
| 21 | PLAU | plasminogen activator, urokinase |
| 22 | PRKCA | protein kinase C alpha |
| 23 | PROM1 | prominin 1 |
| 24 | SEMA4D | semaphorin 4D |
| 25 | STAT3 | signal transducer and activator of transcription 3 |
| 26 | TGFB1 | transforming growth factor beta 1 |
| 27 | TWIST1 | twist family bHLH transcription factor 1 |
| 28 | VEGFA | vascular endothelial growth factor A |
| 29 | WNT5A | Wnt family member 5A |
| 30 | XAF1 | XIAP associated factor 1 |
| 31 | ZEB1 | zinc finger E-box binding homeobox 1 |
| 32 | SNAI1 | snail family transcriptional repressor 1 |
| 33 | SNAI2 | snail family transcriptional repressor 2 |

| **Table S3. Immune Checkpoint Genes List** | | |
| --- | --- | --- |
| **No.** | **Gene symbol** | **Descriptions** |
| 1 | ADORA2A | Adenosine A2a Receptor |
| 2 | BTLA | B And T Lymphocyte Associated |
| 3 | BTN2A1 | Butyrophilin Subfamily 2 Member A1 |
| 4 | BTN2A2 | Butyrophilin Subfamily 2 Member A2 |
| 5 | BTN3A1 | Butyrophilin Subfamily 3 Member A1 |
| 6 | BTNL3 | Butyrophilin Like 3 |
| 7 | BTNL9 | Butyrophilin Like 9 |
| 8 | C10orf54 | V-Set Immunoregulatory Receptor |
| 9 | CD160 | CD160 Molecule |
| 10 | CD209 | CD209 Molecule |
| 11 | CD226 | CD226 Molecule |
| 12 | CD27 | CD27 Molecule |
| 13 | CD274 | CD274 Molecule |
| 14 | CD276 | CD276 Molecule |
| 15 | CD28 | CD28 Molecule |
| 16 | CD40 | CD40 Molecule |
| 17 | CD40LG | CD40 Ligand |
| 18 | CD47 | CD47 Molecule |
| 19 | CD70 | CD70 Molecule |
| 20 | CD80 | CD80 Molecule |
| 21 | CD86 | CD86 Molecule |
| 22 | CD96 | CD96 Molecule |
| 23 | CEACAM1 | CEA Cell Adhesion Molecule 1 |
| 24 | CTLA4 | Cytotoxic T-Lymphocyte Associated Protein 4 |
| 25 | HAVCR2 | Hepatitis A Virus Cellular Receptor 2 |
| 26 | HLA-A | Major Histocompatibility Complex, Class I, A |
| 27 | HLA-B | Major Histocompatibility Complex, Class I, B |
| 28 | HLA-C | Major Histocompatibility Complex, Class I, C |
| 29 | HLA-DMA | Major Histocompatibility Complex, Class II, DM Alpha |
| 30 | HLA-DMB | Major Histocompatibility Complex, Class II, DM Beta |
| 31 | HLA-DOA | Major Histocompatibility Complex, Class II, DO Alpha |
| 32 | HLA-DOB | Major Histocompatibility Complex, Class II, DO Beta |
| 33 | HLA-DPA1 | Major Histocompatibility Complex, Class II, DP Alpha 1 |
| 34 | HLA-DPB1 | Major Histocompatibility Complex, Class II, DP Beta 1 |
| 35 | HLA-DQA1 | Major Histocompatibility Complex, Class II, DQ Alpha 1 |
| 36 | HLA-DQB1 | Major Histocompatibility Complex, Class II, DQ Beta 1 |
| 37 | HLA-DRA | Major Histocompatibility Complex, Class II, DR Alpha |
| 38 | HLA-DRB1 | Major Histocompatibility Complex, Class II, DR Beta 1 |
| 39 | HLA-DRB3 | Major Histocompatibility Complex, Class II, DR Beta 3 |
| 40 | HLA-DRB4 | Major Histocompatibility Complex, Class II, DR Beta 4 |
| 41 | HLA-DRB5 | Major Histocompatibility Complex, Class II, DR Beta 5 |
| 42 | HLA-E | Major Histocompatibility Complex, Class I, E |
| 43 | HLA-F | Major Histocompatibility Complex, Class I, F |
| 44 | HLA-G | Major Histocompatibility Complex, Class I, G |
| 45 | ICOS | Inducible T Cell Costimulator |
| 46 | ICOSLG | Inducible T Cell Costimulator Ligand |
| 47 | IDO1 | Indoleamine 2,3-Dioxygenase 1 |
| 48 | KIR2DL1 | Killer Cell Immunoglobulin Like Receptor, Two Ig Domains And Long Cytoplasmic Tail 1 |
| 49 | KIR2DL2 | Killer Cell Immunoglobulin Like Receptor, Two Ig Domains And Long Cytoplasmic Tail 2 |
| 50 | KIR2DL3 | Killer Cell Immunoglobulin Like Receptor, Two Ig Domains And Long Cytoplasmic Tail 3 |
| 51 | KIR2DL4 | Killer Cell Immunoglobulin Like Receptor, Two Ig Domains And Long Cytoplasmic Tail 4 |
| 52 | KIR2DL5A | Killer Cell Immunoglobulin Like Receptor, Two Ig Domains And Long Cytoplasmic Tail 5A |
| 53 | KIR2DL5B | Killer Cell Immunoglobulin Like Receptor, Two Ig Domains And Long Cytoplasmic Tail 5B |
| 54 | KIR2DS1 | Killer Cell Immunoglobulin Like Receptor, Two Ig Domains And Short Cytoplasmic Tail 1 |
| 55 | KIR2DS2 | Killer Cell Immunoglobulin Like Receptor, Two Ig Domains And Short Cytoplasmic Tail 2 |
| 56 | KIR2DS3 | Killer Cell Immunoglobulin Like Receptor, Two Ig Domains And Short Cytoplasmic Tail 3 |
| 57 | KIR2DS4 | Killer Cell Immunoglobulin Like Receptor, Two Ig Domains And Short Cytoplasmic Tail 4 |
| 58 | KIR2DS5 | Killer Cell Immunoglobulin Like Receptor, Two Ig Domains And Short Cytoplasmic Tail 5 |
| 59 | KIR3DL1 | Killer Cell Immunoglobulin Like Receptor, Three Ig Domains And Long Cytoplasmic Tail 1 |
| 60 | KIR3DL2 | Killer Cell Immunoglobulin Like Receptor, Three Ig Domains And Long Cytoplasmic Tail 2 |
| 61 | KIR3DL3 | Killer Cell Immunoglobulin Like Receptor, Three Ig Domains And Long Cytoplasmic Tail 3 |
| 62 | KIR3DS1 | Killer Cell Immunoglobulin Like Receptor, Three Ig Domains And Short Cytoplasmic Tail 1 |
| 63 | LAG3 | Lymphocyte Activating 3 |
| 64 | LGALS9 | Galectin 9 |
| 65 | PDCD1 | Programmed Cell Death 1 |
| 66 | PDCD1LG2 | Programmed Cell Death 1 Ligand 2 |
| 67 | PVR | PVR Cell Adhesion Molecule |
| 68 | SIRPA | Signal Regulatory Protein Alpha |
| 69 | TDO2 | Tryptophan 2,3-Dioxygenase |
| 70 | TIGIT | T Cell Immunoreceptor With Ig And ITIM Domains |
| 71 | TNFRSF14 | TNF Receptor Superfamily Member 14 |
| 72 | TNFRSF18 | TNF Receptor Superfamily Member 18 |
| 73 | TNFRSF4 | TNF Receptor Superfamily Member 4 |
| 74 | TNFRSF9 | TNF Receptor Superfamily Member 9 |
| 75 | TNFSF14 | TNF Superfamily Member 14 |
| 76 | TNFSF18 | TNF Superfamily Member 18 |
| 77 | TNFSF4 | TNF Superfamily Member 4 |
| 78 | TNFSF9 | TNF Superfamily Member 9 |
| 79 | VTCN1 | V-Set Domain Containing T Cell Activation Inhibitor 1 |

| **Table S4. The clinical characteristics of patients for IHC-PAS** | | | |
| --- | --- | --- | --- |
| **Characteristics** | **VM (+)** | **VM (-)** | ***p*** |
| Age | 59.29±6.448 | 54.17±9.270 | 0.142 |
| Grade | 3 | 3 | 1 |
| FIGO Stage | 3 | 3 | 0.387 |
| Maximum diameter of the primary lesion | 93.86±46.785 | 104.03±40.124 | 0.667 |

| **Table S5. qRT-PCR Primer** | | |
| --- | --- | --- |
| **Gene symbol** | **Forward (5’→3’)** | **Reverse (5’→3’)** |
| hGAPDH | GAAGGTGAAGGTCGGAGTC | GAAGATGGTGATGGGATTTC |
| SNAI1 | TCGGAAGCCTAACTACAGCGA | AGATGAGCATTGGCAGCGAG |
| SNAI2 | CGAACTGGACACACATACAGTG | CTGAGGATCTCTGGTTGTGGT |
| MMP2 | TGGCAAGTACGGCTTCTGTC | TTCTTGTCGCGGTCGTAGTC |
| MMP14 | CAAGATTGATGCTGCTCTCTTC | ACTTTGATGTTCTTGGGGTACT |
| ZEB1 | GATGATGAATGCGAGTCAGATGC | ACAGCAGTGTCTTGTTGTTGT |
| TWIST1 | GTCCGCAGTCTTACGAGGAG | GCTTGAGGGTCTGAATCTTGCT |
| FPR1 | CTCCAGTTGGACTAGCCACA | CCATCACCCAGGGCCCAATG |
| ADH1B | AGGGTAGAGGAGGCTGAAGA | ACCTGCTTCACTCTGGGAAA |
| RARRES1 | TGGCTTTCCTTGGAAGCTCT | AGGTTTTTCTTACCCACTGCCT |
| TSPAN8 | TGCCTGGAGATAGCCTTTGC | ACCACATAGCCAGAACAAGAAG |
| FOXJ1 | TCGTATGCCACGCTCATCTG | CGGATTGAATTCTGCCAGGT |
| CXCL13 | GCTTGAGGTGTAGATGTGTCC | CCCACGGGGCAAGATTTGAA |
| WNT11 | ATTTGCTTGACCTGGAGAGAGG | TGAGGTTGTCCGCACATCC |
| CXCL9 | CCAGTAGTGAGAAAGGGTCGC | AGGGCTTGGGGCAAATTGTT |
| SST | AACCCAACCAGACGGAGAA | TAGCCGGGTTTGAGTTAGCA |

| **Table S6. The Complete Gene List for Model Construction** | | | | |
| --- | --- | --- | --- | --- |
| **Symbol** | **HR** | **HR.95L** | **HR.95H** | ***p*-value** |
| FPR1 | 1.26001399 | 1.12124301 | 1.41596001 | 0.000103522 |
| CYTH3 | 1.38199061 | 1.1702525 | 1.63203929 | 0.000137375 |
| KIF26B | 1.2218034 | 1.09921333 | 1.35806535 | 0.000204448 |
| MFAP4 | 1.13666929 | 1.06227137 | 1.21627779 | 0.000208043 |
| TEKT2 | 0.83852539 | 0.76392025 | 0.92041654 | 0.000211994 |
| GFPT2 | 1.25237171 | 1.11142325 | 1.41119497 | 0.000220656 |
| EMP1 | 1.2460411 | 1.10389577 | 1.40649005 | 0.000371696 |
| VAT1L | 1.36278035 | 1.14809358 | 1.61761228 | 0.00040172 |
| CH25H | 1.27470756 | 1.11418438 | 1.4583577 | 0.000408626 |
| C5AR1 | 1.25730479 | 1.10566358 | 1.42974353 | 0.000479901 |
| PTGER3 | 1.22350296 | 1.09120297 | 1.3718433 | 0.000550664 |
| SVEP1 | 1.22529666 | 1.08893424 | 1.37873515 | 0.000737318 |
| LRRN4 | 1.17078796 | 1.06756835 | 1.28398751 | 0.000812615 |
| DKK2 | 1.27483722 | 1.10193468 | 1.47486958 | 0.001093626 |
| FOXJ1 | 0.89414399 | 0.83577022 | 0.95659482 | 0.001161298 |
| RASSF2 | 1.26984523 | 1.09912333 | 1.4670846 | 0.001182944 |
| SH3PXD2A | 1.23601161 | 1.0872609 | 1.40511325 | 0.001200635 |
| FLNC | 1.14964097 | 1.05645678 | 1.25104442 | 0.001223254 |
| SPDEF | 0.89759992 | 0.83978507 | 0.95939503 | 0.00147145 |
| CXCL13 | 0.88599532 | 0.82202111 | 0.95494837 | 0.00154811 |
| EPB41L3 | 1.31010126 | 1.10744973 | 1.54983587 | 0.001630894 |
| TIMP3 | 1.13962561 | 1.05058025 | 1.23621829 | 0.00164015 |
| SLIT3 | 1.16814119 | 1.06042628 | 1.28679746 | 0.001640485 |
| PLAUR | 1.25348476 | 1.08901926 | 1.44278812 | 0.001642171 |
| GALNT10 | 1.29782955 | 1.10321733 | 1.52677218 | 0.001660701 |
| SYDE1 | 1.23903168 | 1.08299359 | 1.41755179 | 0.001802866 |
| FAM81B | 0.86160067 | 0.78412483 | 0.94673155 | 0.001944277 |
| PHLDB2 | 1.23547966 | 1.08013034 | 1.41317204 | 0.002040732 |
| ADH1B | 1.1622831 | 1.05630943 | 1.27888851 | 0.002049169 |
| CD163 | 1.17380883 | 1.06003923 | 1.29978885 | 0.002063734 |
| MEOX2 | 1.2429969 | 1.08158019 | 1.42850368 | 0.002177038 |
| FAP | 1.14768241 | 1.05090248 | 1.25337502 | 0.002179814 |
| FSTL3 | 1.21632171 | 1.07312863 | 1.37862178 | 0.00218124 |
| TUBB2A | 1.22170613 | 1.07355005 | 1.39030859 | 0.0023978 |
| PTGIS | 1.13964992 | 1.04565115 | 1.24209871 | 0.002916957 |
| PTGIR | 1.30222075 | 1.09384307 | 1.55029449 | 0.002995862 |
| DACT3 | 1.22147851 | 1.06885656 | 1.39589334 | 0.003305612 |
| TGFBI | 1.16237277 | 1.05122379 | 1.28527387 | 0.003345043 |
| C1QTNF3 | 1.16916321 | 1.05308915 | 1.29803124 | 0.003394061 |
| SNAI1 | 1.21470354 | 1.0654336 | 1.38488657 | 0.003644464 |
| SCG2 | 1.1539861 | 1.04775882 | 1.27098326 | 0.003650943 |
| TMEM119 | 1.15429523 | 1.04729037 | 1.2722331 | 0.003841704 |
| ADAMTSL1 | 1.27616852 | 1.08101652 | 1.5065506 | 0.003977116 |
| OLFML3 | 1.17249708 | 1.05180571 | 1.30703741 | 0.004088032 |
| FGF7 | 1.20731509 | 1.06161352 | 1.37301353 | 0.004089876 |
| PODN | 1.14934475 | 1.04478088 | 1.2643736 | 0.004234909 |
| MS4A7 | 1.20839072 | 1.06042663 | 1.37700063 | 0.004506491 |
| PTPRD | 1.21923792 | 1.0632309 | 1.39813574 | 0.004544427 |
| CXCL12 | 1.12916444 | 1.03805113 | 1.22827508 | 0.004655442 |
| EPHB2 | 1.21350137 | 1.06120659 | 1.38765211 | 0.004680905 |
| GFRA1 | 1.15428616 | 1.04483787 | 1.27519932 | 0.004758935 |
| RCAN1 | 1.22834403 | 1.06414386 | 1.41788072 | 0.004967571 |
| GAS1 | 1.14823906 | 1.0420948 | 1.26519482 | 0.005219945 |
| SH3PXD2B | 1.20314982 | 1.05650706 | 1.37014655 | 0.005289364 |
| WNT11 | 1.10068624 | 1.02892192 | 1.17745592 | 0.005290454 |
| DSE | 1.32271464 | 1.08605467 | 1.61094471 | 0.005423963 |
| SPOCK1 | 1.14079174 | 1.03964031 | 1.25178466 | 0.005426032 |
| GPR34 | 1.16891552 | 1.04701878 | 1.30500379 | 0.005474922 |
| ITGBL1 | 1.21840776 | 1.0597706 | 1.40079134 | 0.005509302 |
| RARRES1 | 1.09811561 | 1.02763858 | 1.17342607 | 0.005682869 |
| NKX3-2 | 1.17254269 | 1.047398 | 1.31263987 | 0.005707288 |
| LOX | 1.13251083 | 1.03677514 | 1.23708675 | 0.005755468 |
| MFAP5 | 1.09782847 | 1.02739996 | 1.17308489 | 0.005797311 |
| MAFB | 1.19125618 | 1.05198719 | 1.34896251 | 0.005799177 |
| CILP2 | 1.15634031 | 1.04291485 | 1.28210172 | 0.005821298 |
| TGFB3 | 1.17902781 | 1.04860916 | 1.32566701 | 0.005895199 |
| SFRP2 | 1.06475286 | 1.01795075 | 1.11370679 | 0.006224553 |
| ITGA5 | 1.18464591 | 1.04879124 | 1.33809845 | 0.006400843 |
| ZCCHC24 | 1.18435286 | 1.04804543 | 1.33838825 | 0.006683903 |
| CXCL9 | 0.91728631 | 0.86178795 | 0.97635872 | 0.006701547 |
| CRISPLD2 | 1.13528864 | 1.03568626 | 1.24446983 | 0.006760563 |
| CXCL14 | 1.08403805 | 1.0224859 | 1.14929555 | 0.006819404 |
| NTM | 1.1410097 | 1.036767 | 1.25573358 | 0.006962566 |
| SEMA3D | 1.19268643 | 1.04937208 | 1.35557345 | 0.006980023 |
| VEGFC | 1.19045505 | 1.04831238 | 1.35187113 | 0.007204825 |
| CTHRC1 | 1.11697308 | 1.0300782 | 1.21119819 | 0.00742521 |
| AQP1 | 1.19694308 | 1.04916195 | 1.36554012 | 0.007500856 |
| SCGB1D2 | 0.91356674 | 0.85479946 | 0.97637427 | 0.007704399 |
| STARD8 | 1.30839107 | 1.07202899 | 1.59686651 | 0.008189529 |
| OSM | 1.16937298 | 1.04086844 | 1.31374255 | 0.008429783 |
| PRG4 | 1.15578603 | 1.03740504 | 1.28767578 | 0.008638541 |
| EGR2 | 1.14283404 | 1.03422211 | 1.26285218 | 0.008782743 |
| NUAK1 | 1.16374129 | 1.03880695 | 1.30370113 | 0.008869773 |
| LAMA4 | 1.18597226 | 1.04364458 | 1.34770996 | 0.008925746 |
| KLF6 | 1.20216609 | 1.04714135 | 1.38014157 | 0.008951422 |
| SST | 0.94714228 | 0.90919257 | 0.98667601 | 0.009244625 |
| MEIS3 | 1.18274327 | 1.04189916 | 1.34262672 | 0.00947431 |
| BNC1 | 1.1388762 | 1.03205823 | 1.25674982 | 0.009655342 |
| GLIPR1 | 1.23531095 | 1.05251685 | 1.44985152 | 0.009697921 |
| LRRC17 | 1.13701975 | 1.03130636 | 1.25356921 | 0.009905655 |
| DPT | 1.19008925 | 1.04246405 | 1.35861993 | 0.010012346 |
| STAB1 | 1.15849676 | 1.03516367 | 1.2965242 | 0.010415639 |
| ELN | 1.12100444 | 1.02718774 | 1.22338974 | 0.010421781 |
| UNC5B | 1.1484656 | 1.03293809 | 1.27691412 | 0.010495225 |
| CD14 | 1.15508836 | 1.03376965 | 1.29064451 | 0.010878286 |
| HSPB7 | 1.15858706 | 1.03448573 | 1.29757612 | 0.010881253 |
| C9orf24 | 0.91347038 | 0.85195254 | 0.9794303 | 0.010951366 |
| FBLN1 | 1.13275988 | 1.02900358 | 1.24697811 | 0.010981618 |
| OMD | 1.12650804 | 1.02765837 | 1.23486598 | 0.01101543 |
| THBS2 | 1.08801951 | 1.01949939 | 1.16114484 | 0.011026694 |
| FMO2 | 1.14410946 | 1.03125234 | 1.26931731 | 0.011061609 |
| COL5A2 | 1.10006461 | 1.02198655 | 1.18410772 | 0.011118293 |
| P4HA3 | 1.26802191 | 1.05520466 | 1.52376086 | 0.011302419 |
| ALDH1A2 | 1.10476913 | 1.02280064 | 1.19330666 | 0.011304822 |
| LSAMP | 1.23287345 | 1.04823999 | 1.45002762 | 0.011434514 |
| EDNRA | 1.15200735 | 1.03235214 | 1.28553124 | 0.01143852 |
| PLK2 | 1.16025188 | 1.03396691 | 1.30196083 | 0.011468323 |
| MEGF10 | 1.21485486 | 1.04424747 | 1.4133358 | 0.011711183 |
| ASPN | 1.10603822 | 1.02265302 | 1.19622249 | 0.011733058 |
| MOXD1 | 1.1309834 | 1.02760613 | 1.24476044 | 0.011843373 |
| TMEM45A | 1.17298267 | 1.03568845 | 1.32847707 | 0.012002316 |
| FN1 | 1.09166181 | 1.0194503 | 1.16898833 | 0.012016903 |
| JAM2 | 1.19854883 | 1.04044358 | 1.38067968 | 0.012098163 |
| FBN1 | 1.12769864 | 1.02661775 | 1.23873196 | 0.012133651 |
| MAF | 1.17621972 | 1.03570541 | 1.33579761 | 0.012403915 |
| MSR1 | 1.17839002 | 1.03557641 | 1.34089867 | 0.01276292 |
| GPC1 | 1.18636082 | 1.03673785 | 1.35757751 | 0.012973264 |
| CD36 | 1.16913894 | 1.03333428 | 1.32279156 | 0.013121452 |
| NT5E | 1.12205648 | 1.0243721 | 1.22905606 | 0.013207568 |
| RGS16 | 1.14060008 | 1.02743232 | 1.26623284 | 0.01360282 |
| ADAMTS2 | 1.12120925 | 1.02368308 | 1.22802673 | 0.013735754 |
| COL8A2 | 1.12842989 | 1.02494933 | 1.24235801 | 0.0138117 |
| RGS2 | 1.13438495 | 1.02587824 | 1.25436837 | 0.01397057 |
| ZFP36 | 1.12826895 | 1.02453787 | 1.24250246 | 0.014182174 |
| BMP4 | 1.12889628 | 1.02458616 | 1.24382592 | 0.014246651 |
| ALOX5AP | 1.12197043 | 1.02314486 | 1.23034156 | 0.014431656 |
| FCGR2A | 1.16286361 | 1.03039472 | 1.31236287 | 0.014477539 |
| RSPH1 | 0.87006327 | 0.77800418 | 0.97301544 | 0.014712332 |
| GJB2 | 1.09077145 | 1.01706965 | 1.16981403 | 0.014926976 |
| COL5A1 | 1.09018447 | 1.01693864 | 1.16870588 | 0.014961599 |
| CLIC3 | 1.12378104 | 1.02285098 | 1.2346704 | 0.015076591 |
| DES | 1.09158163 | 1.01689417 | 1.17175464 | 0.015382247 |
| HBEGF | 1.18193736 | 1.03246377 | 1.3530508 | 0.015389434 |
| SIRPA | 1.16652622 | 1.02964657 | 1.32160245 | 0.01557452 |
| THY1 | 1.13064981 | 1.02344164 | 1.24908832 | 0.015699167 |
| ROPN1L | 0.90002016 | 0.82627634 | 0.98034549 | 0.015732351 |
| GEM | 1.15405363 | 1.02723082 | 1.2965341 | 0.015852436 |
| SLCO2B1 | 1.13136179 | 1.02340218 | 1.25071015 | 0.015863329 |
| ADRA2A | 1.11104248 | 1.01973133 | 1.21053003 | 0.016105491 |
| PXDN | 1.12799175 | 1.02254438 | 1.24431312 | 0.016164583 |
| NGFR | 1.12917322 | 1.02242193 | 1.24707043 | 0.016503567 |
| TNFAIP6 | 1.14106702 | 1.02414583 | 1.27133647 | 0.016732597 |
| CALB2 | 1.08967601 | 1.01523386 | 1.16957665 | 0.017372264 |
| TSPAN8 | 0.90654881 | 0.83597883 | 0.98307604 | 0.017655375 |
| COL16A1 | 1.13519513 | 1.02223936 | 1.26063232 | 0.017726069 |
| DDIT4L | 1.12024192 | 1.01951984 | 1.23091471 | 0.018170092 |
| BGN | 1.12848407 | 1.0205671 | 1.24781242 | 0.018426675 |
| MATN3 | 1.17051849 | 1.02651357 | 1.3347252 | 0.018740664 |
| COL11A1 | 1.07152216 | 1.01140586 | 1.13521167 | 0.019029751 |
| CSF1R | 1.1249844 | 1.01927161 | 1.2416611 | 0.019330874 |
| LRP1 | 1.14912619 | 1.02237017 | 1.29159773 | 0.01975539 |
| IGFBP4 | 1.14873161 | 1.02227847 | 1.29082667 | 0.019792353 |
| VCAN | 1.098779 | 1.01498956 | 1.18948543 | 0.019933596 |
| GADD45B | 1.15689076 | 1.02322166 | 1.30802178 | 0.019996248 |
| RUNX1 | 1.15795263 | 1.02329214 | 1.31033382 | 0.020072238 |
| KLF2 | 1.1423138 | 1.02091991 | 1.27814221 | 0.020279042 |
| FZD1 | 1.15965998 | 1.02304141 | 1.31452281 | 0.020549602 |
| IGLON5 | 1.08890115 | 1.01286029 | 1.17065081 | 0.021114348 |
| NBL1 | 1.13451032 | 1.01841892 | 1.26383519 | 0.021943751 |
| DUSP1 | 1.1068642 | 1.01472851 | 1.20736566 | 0.022039042 |
| EMCN | 1.24053249 | 1.03136969 | 1.49211372 | 0.022148253 |
| OGN | 1.10517844 | 1.0140491 | 1.20449729 | 0.02274364 |
| LRRC15 | 1.0879936 | 1.0118275 | 1.16989316 | 0.022756716 |
| BCHE | 1.14159881 | 1.01863206 | 1.27940981 | 0.022760142 |
| MMP2 | 1.10146499 | 1.01349429 | 1.19707149 | 0.022870795 |
| MFAP2 | 1.08957258 | 1.01176098 | 1.17336845 | 0.023252603 |
| MORN5 | 0.90629059 | 0.83232578 | 0.98682829 | 0.02349968 |
| MMP14 | 1.13295632 | 1.01695888 | 1.26218479 | 0.023505858 |
| SLC2A3 | 1.13129553 | 1.016525 | 1.25902421 | 0.023805129 |
| SLC12A8 | 1.18539574 | 1.02245098 | 1.37430849 | 0.024180793 |
| CAPSL | 0.9216437 | 0.858517 | 0.98941209 | 0.024196123 |
| OLR1 | 1.13663502 | 1.01675545 | 1.27064887 | 0.024311526 |
| KLF10 | 1.18419498 | 1.02211026 | 1.37198285 | 0.024375006 |
| SLCO2A1 | 1.14566258 | 1.01769292 | 1.28972376 | 0.024437695 |
| COMP | 1.06665441 | 1.00832892 | 1.12835367 | 0.024508501 |
| GLT8D2 | 1.13043488 | 1.0158704 | 1.25791933 | 0.024526937 |
| COL8A1 | 1.10450683 | 1.01278272 | 1.20453806 | 0.0246329 |
| COL3A1 | 1.08231421 | 1.01001523 | 1.15978849 | 0.024930565 |
| ADAM12 | 1.10106331 | 1.01184424 | 1.19814925 | 0.025544368 |
| FBLN2 | 1.09219286 | 1.01078249 | 1.18016018 | 0.025659762 |
| PALLD | 1.15414701 | 1.0173813 | 1.30929804 | 0.025898091 |
| ENPP1 | 1.15306728 | 1.01677119 | 1.30763358 | 0.026479543 |
| FAM20C | 1.14403298 | 1.01480733 | 1.28971424 | 0.027784345 |
| LUM | 1.06994969 | 1.00741204 | 1.13636953 | 0.027786719 |
| HOXA5 | 1.08522303 | 1.00882477 | 1.16740693 | 0.0281019 |
| AOC3 | 1.13654795 | 1.01358919 | 1.27442286 | 0.028450624 |
| RGS4 | 1.13117494 | 1.01298583 | 1.26315364 | 0.028588446 |
| ALDH1A3 | 1.1705676 | 1.01632082 | 1.34822438 | 0.028923903 |
| CYP26B1 | 1.13718927 | 1.01304096 | 1.27655199 | 0.029284564 |
| SERPINF1 | 1.09692135 | 1.00928185 | 1.19217089 | 0.029448382 |
| HSD17B6 | 1.18699618 | 1.01725736 | 1.38505749 | 0.029459691 |
| SLC7A7 | 1.16160958 | 1.01469417 | 1.32979656 | 0.029900808 |
| AOAH | 1.13793775 | 1.01255623 | 1.27884486 | 0.030047741 |
| SLIT2 | 1.13946382 | 1.01257611 | 1.28225206 | 0.03020108 |
| OLFML2B | 1.10668112 | 1.00968678 | 1.2129931 | 0.030314824 |
| SNAI2 | 1.11363117 | 1.01010724 | 1.22776506 | 0.030619881 |
| OSR2 | 1.10986521 | 1.00943552 | 1.22028673 | 0.03123786 |
| GAL3ST4 | 1.17184662 | 1.01420709 | 1.35398827 | 0.031449029 |
| VGLL3 | 1.16755178 | 1.0129541 | 1.34574425 | 0.032551831 |
| NR4A3 | 1.13521084 | 1.00993538 | 1.27602585 | 0.033530361 |
| GPX3 | 1.0832963 | 1.00608541 | 1.16643265 | 0.03394025 |
| DDR2 | 1.14002577 | 1.00996657 | 1.28683346 | 0.033970095 |
| ACSL1 | 1.15843051 | 1.01100845 | 1.32734918 | 0.034208203 |
| SDK1 | 1.12337914 | 1.00864829 | 1.2511603 | 0.034291414 |
| MMP19 | 1.13959381 | 1.00957811 | 1.28635322 | 0.03449864 |
| POSTN | 1.0584089 | 1.00414222 | 1.1156083 | 0.034524277 |
| C5orf46 | 1.11956439 | 1.0079244 | 1.24356989 | 0.035097207 |
| SORCS2 | 1.11025963 | 1.00693319 | 1.2241889 | 0.035852797 |
| FOSB | 1.06714054 | 1.00427803 | 1.13393792 | 0.035925216 |
| SLC2A5 | 1.16250888 | 1.00991111 | 1.3381642 | 0.035964442 |
| PRRX1 | 1.09347478 | 1.00579794 | 1.18879454 | 0.036123463 |
| CYTH4 | 1.1601104 | 1.00951769 | 1.33316746 | 0.036305228 |
| THBD | 1.13400584 | 1.00756542 | 1.2763134 | 0.037076621 |
| GREM1 | 1.1316173 | 1.00716529 | 1.27144742 | 0.037518866 |
| TNFSF4 | 1.14728242 | 1.00771319 | 1.30618213 | 0.037888071 |
| CACNA1G | 1.19355979 | 1.00971269 | 1.41088152 | 0.038151909 |
| PMP22 | 1.15048371 | 1.00709877 | 1.31428297 | 0.039006263 |
| COLEC12 | 1.11976589 | 1.00572065 | 1.24674346 | 0.039013079 |
| FSTL1 | 1.12478511 | 1.00588144 | 1.2577442 | 0.039128231 |
| LZTS1 | 1.14528899 | 1.00675792 | 1.30288209 | 0.039173735 |
| SPARC | 1.10652846 | 1.00499304 | 1.21832212 | 0.039265974 |
| SCT | 1.15424894 | 1.00693715 | 1.323112 | 0.039474897 |
| ZEB1 | 1.17396372 | 1.0076508 | 1.36772662 | 0.039614123 |
| RARRES2 | 1.10959864 | 1.00447609 | 1.2257227 | 0.040568889 |
| FRMD6 | 1.16934789 | 1.00663635 | 1.35835994 | 0.040708393 |
| PDPN | 1.09712835 | 1.00383567 | 1.19909129 | 0.04091432 |
| LHFPL2 | 1.20205633 | 1.00670273 | 1.43531888 | 0.041972784 |
| ZEB2 | 1.21199017 | 1.00674333 | 1.45908111 | 0.042259328 |
| ENOX1 | 1.1937337 | 1.00602665 | 1.41646361 | 0.0424785 |
| LATS2 | 1.18663518 | 1.00580345 | 1.39997833 | 0.042502908 |
| DAB2 | 1.15517395 | 1.00478479 | 1.32807232 | 0.042658287 |
| TUBB6 | 1.14703505 | 1.00413188 | 1.31027551 | 0.043310603 |
| CPA3 | 1.15244845 | 1.00410919 | 1.32270221 | 0.043560517 |
| COL1A1 | 1.07716705 | 1.00213197 | 1.15782041 | 0.043614833 |
| OVGP1 | 0.94385496 | 0.89225967 | 0.99843376 | 0.043945101 |
| CILP | 1.09052541 | 1.00233314 | 1.18647745 | 0.04399618 |
| CMTM3 | 1.19431171 | 1.00462405 | 1.41981516 | 0.044194281 |
| LAPTM5 | 1.10292871 | 1.00235403 | 1.2135949 | 0.044626305 |
| INHBA | 1.08026976 | 1.00184401 | 1.16483478 | 0.04465674 |
| RAI14 | 1.1513972 | 1.00333097 | 1.32131426 | 0.044716257 |
| ADAMTS10 | 1.13999317 | 1.00305627 | 1.29562465 | 0.044781444 |
| MXRA8 | 1.08778122 | 1.00195588 | 1.18095816 | 0.044796329 |
| EPYC | 1.05622006 | 1.00118361 | 1.11428195 | 0.045146323 |
| COLEC11 | 0.93061672 | 0.86732127 | 0.99853135 | 0.045407517 |
| F13A1 | 1.09901006 | 1.00170002 | 1.20577326 | 0.04594777 |
| MS4A4A | 1.13043045 | 1.00213874 | 1.27514578 | 0.046072881 |
| PCDH12 | 1.2126512 | 1.00306655 | 1.46602728 | 0.046415953 |
| CHSY3 | 1.17510115 | 1.00253505 | 1.37737101 | 0.046457983 |
| CD93 | 1.16097309 | 1.00220098 | 1.34489842 | 0.046671887 |
| FBLN5 | 1.12792796 | 1.00173692 | 1.27001558 | 0.046741863 |
| PROCR | 1.16598188 | 1.0014473 | 1.35754897 | 0.047861254 |
| TWIST1 | 1.09552095 | 1.00078524 | 1.19922448 | 0.048044602 |
| TGFBR2 | 1.16619578 | 1.00122653 | 1.35834655 | 0.048186981 |
| MMP13 | 1.06972326 | 1.00021881 | 1.14405752 | 0.049258558 |
| FERMT2 | 1.20172959 | 1.00031356 | 1.44370133 | 0.049609758 |
| LAIR1 | 1.13653068 | 1.00002379 | 1.29167127 | 0.049957429 |
| ZFHX4 | 1.42752791 | 1.2147645 | 1.67755638 | 1.54217526819674e-05 |
| CCDC80 | 1.2020756 | 1.09966871 | 1.31401914 | 5.0933847193391e-05 |
| DOCK11 | 1.32010881 | 1.15114158 | 1.51387744 | 7.06092853446442e-05 |
| VSIG4 | 1.21641714 | 1.10274385 | 1.34180813 | 9.08553432616087e-05 |

**Supplementary Figure**


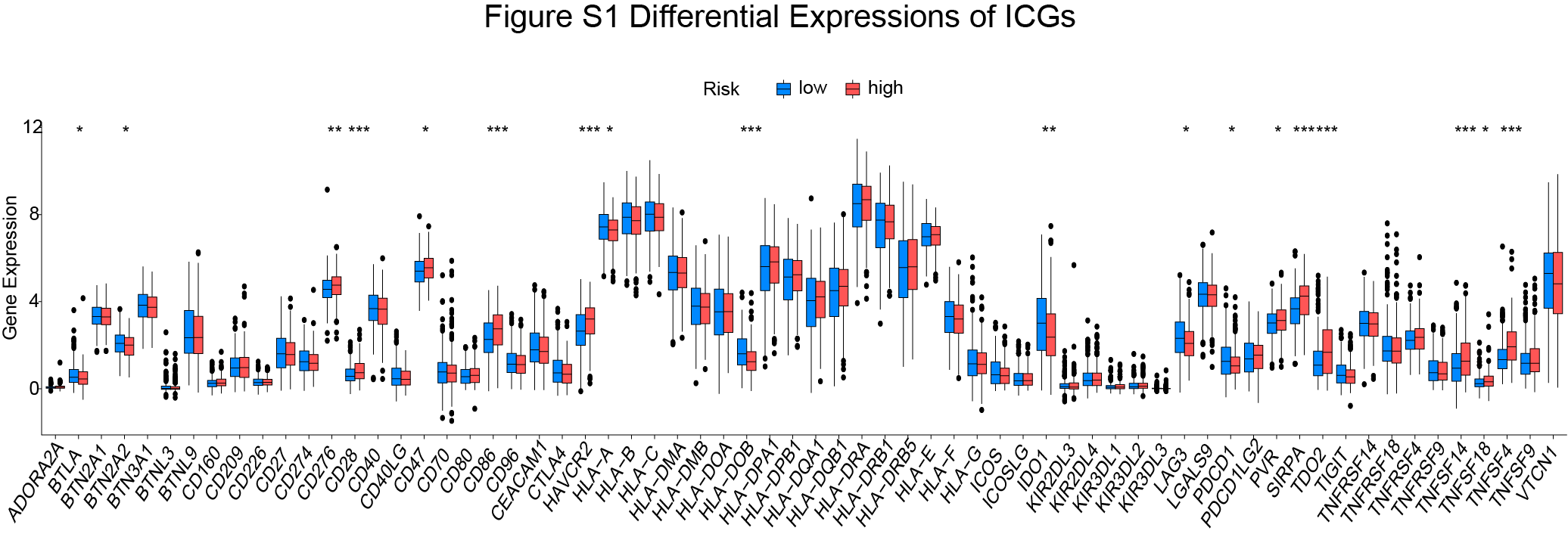


Figure S1: Different Expression of ICGs

The expression levels of immune checkpoint genes (ICGs) were compared between the high-risk group and low-risk group. In results’ figures, * means p < 0.05, ** means p < 0.01, and *** means p < 0.001. Non statistically significant results are indicated by ns.
